# Design and implementation of a Li River water quality monitoring and analysis system based on outlier data analysis

**DOI:** 10.1371/journal.pone.0299435

**Published:** 2024-03-18

**Authors:** Qirong Lu, Jian Zou, Yingya Ye, Zexin Wang

**Affiliations:** 1 College of Information Science and Engineering, Guilin University of Technology, Guilin, China; 2 Guangxi Key Laboratory of Embedded Technology and Intelligent System, Guilin University of Technology, Guilin, China; Sunway University, MALAYSIA

## Abstract

The detection of water quality indicators such as Temperature, pH, Turbidity, Conductivity, and TDS involves five national standard methods. Chemically based measurement techniques may generate liquid residue, causing secondary pollution. The water quality monitoring and data analysis system can effectively address the issues that conventional methods require multiple pieces of equipment and repeated measurements. This paper analyzes the distribution characteristics of the historical data from five sensors at a specific time, displays them graphically in real time, and provides an early warning of exceeding the standard; It selects four water samples from different sections of the Li River, based on the national standard method, the average measurement errors of Temperature, PH, TDS, Conductivity and Turbidity are 0.98%, 2.23%, 2.92%, 3.05% and 3.98%.;It further uses the quartile method to analyze the outlier data over 100,000 records and five historical periods are selected. Experiment results show the system is relatively stable in measuring Temperature, PH and TDS, and the proportion of outlier is 0.42%, 0.84% and 1.24%. When Turbidity and Conductivity are measured, the proportion is 3.11% and 2.92%. In the experiment of using 7 methods to fill outlier, K nearest neighbor algorithm is better than others. The analysis of data trends, outliers, means, and extreme values assists in making decisions, such as updating and maintaining equipment, addressing extreme water quality situations, and enhancing regional water quality oversight.

## Introduction

The water quality in rivers is a critical issue that affects human health and well-being. Chemicals from both human activities and natural sources are entering freshwater lakes and water supply systems, resulting in a decline in water quality and posing risks to human health [1]. According to a report from the World Health Organization, about 159 million people worldwide rely on unsafe surface water sources, posing significant health risks [2]. Human activities contributing to water quality issues include discharges from septic tanks, improper waste disposal, emissions from garbage, mineral extraction, and the excessive use of fertilizers and pesticides in agriculture. Human activities have become major contributors to water pollution, with approximately 50% of untreated wastewater being discharged directly into rivers or oceans, leading to severe ecological damage and long-term consequences [3]. Seasonal rainfall and other natural processes can lead to increased river pollution through surface runoff, which contributes to the deterioration of water quality. Therefore, monitoring water quality is of the utmost importance. This study monitors the Li River, which is a true gem in Guilin’s natural landscape, and its breathtaking views have long been renowned worldwide. Recognizing the importance of this natural beauty, stating that the Li River is a natural heritage shared by all humanity, we must take good care of it. To further fortify the accomplishments of previous governance efforts, it is imperative to conduct water quality monitoring and analysis of the Li River. This undertaking holds considerable implications for the continued preservation of the river’s ecological integrity and the sustainability of the surrounding ecosystem.

The monitoring and assessment of water quality represents a multifaceted process that encompasses a range of indicators. These indicators include temperature, pH, conductivity, TDS (Total Dissolved Solids), turbidity, total alkalinity, organic substances (such as ammonia nitrogen, dissolved oxygen, chemical oxygen demand, nitrate, and phosphate), and Escherichia coli, among others. Scholars select water quality parameters for their studies based on their research requirements. The principal indicators of water quality incorporate PH, Temperature, Chemical oxygen demand, Turbidity, Conductivity, and TDS. A partial analysis of these indicators is provided herein, along with the associated national measurement standards as displayed in Table 1.

**Table 1:** Emission limit values for indicators of rural domestic sewage.

No	Indicators	National standard	Description
1	Temperature	GB 3838–2002	Human-induced changes in environmental water temperature should be limited to:
			Weekly average maximum temperature increase ≤1°C,
			Weekly average maximum temperature decrease ≤2°C.
2	PH	HJ1147-2020	The PH range deemed acceptable for surface water is typically between 6 and 9, whereas the PH range commonly observed in urban drinking water falls within the interval of 6.5 to 8.5.
3	Turbidity	HJ 1075–2019	1. According to the national standard for potable water, the turbidity must not surpass 1 NTU, although in cases where the water source and water treatment technology impose restrictions, a maximum of 3 NTU is allowed.
			2. Small water treatment facilities should be a minimum of turbidity less than 5 NTU, preferably less than 1 NTU [4].
			3. When the turbidity exceeds 1 but is less than 5, it should be set to moderate, and when it exceeds 5, it should be set to high.
4	Conductivity	JJG 376–2007	1. Conductivity is deemed a significant parameter as it can serve as an indicator for several other parameters, including total dissolved solids, salinity, and the total ion concentration present in the solution.
			2. When the conductivity value is less than 1200 μS/cm, it is of good quality. When the value is between 1200 and 2000 μS/cm, it is considered moderate. If the value exceeds 2000 μS/cm, it is considered excessive [4].
5	TDS	GB5749-2022	1. TDS can be cross-validated with the conductivity parameter.
			2. When the TDS is less than 600 mg/L, it is of good quality. When the value is between 600 and 1,000 mg/L, it is considered moderate. If the value exceeds 1,000 mg/L, it is considered excessive [4].

The national standard GB 3838–2002 prescribes a method for measuring water temperature in bodies of water such as rivers and lakes. In compliance with the national standard HJ1147-2020, the glass electrode method is utilized to measure PH, and water turbidity is measured using the turbidimetric method in accordance with the national standard HJ 1075–2019. The methodology for measuring conductivity is specified in the national standard JJG 376–2007. Additionally, the maximum permissible value for TDS in water samples is specified in GB5749-2022. The national standard HJ/T 91–2022 delineates the environmental quality monitoring items, analysis techniques, and data handling procedures for surface water. We have provided detailed supplements for indicators such as TDS, conductivity, and turbidity based on the standards set by the World Health Organization [4].

In the context of organic water quality indicators, many scholars consider indirect methods employing artificial intelligence and machine learning [5–7], such as artificial neural networks, extreme learning machines, random forests, and swarm intelligence algorithms. For example, the measurement of biochemical oxygen demand (BOD5) involves determining the amount of molecular oxygen consumed in 1 liter of water at 20°C over a 5-day incubation period, making the process time-consuming. Therefore, based on historical data, optimization techniques such as genetic algorithms are used in combination with linear regression and multilayer perceptron models [8]. Similarly, spectral techniques are primarily used for the measurement of chemical oxygen demand (COD) [9], making online monitoring challenging. Analyzing literature [5–9] reveals that, for organic water quality indicators, due to cost and measurement accuracy issues, and online monitoring methods are not widely adopted.

By analyzing the monitoring of chemical elements from rivers, estuaries, and seas, literature [10] examined at the relationship between silicate and phytoplankton composition. The research findings indicated that seasonal variations led to changes in physicochemical and nutritional factors, which in turn were responsible for the succession of plankton. The reference [11] reviewed the scope, techniques, and technological requirements for monitoring surface water quality in China, as well as the long-term advancements in the field. Computer technology, Internet of Things (IoT) technology, and wireless sensor network technology are widely used in the field of water quality monitoring. This has significantly advanced research on water quality monitoring. The monitoring of water quality involves the tracking of physical, biochemical, and other indicators and data analysis. The STM32 microcontroller [12–18] combined with a range of sensors is extensively utilized in monitoring systems, which facilitate the acquisition of environmental data. The International Atomic Energy Agency (IAEA) set up a network of monitoring stations as cited in literature [19] to collect continuous, long-term data from river water. Nonetheless, there is a lack of extensive records for Indian rivers. Governments can undertake regional groundwater management measures, and freshwater resources are vital. To preserve freshwater supplies and improve water quality, methods such as artificial recharge, rainwater collection, and routine disinfection can be used [20]. Twenty groundwater samples were randomly collected from open wells for analysis. It was discovered that most groundwater samples were unsuitable for irrigation due to conductivity concentration levels that exceeded drinking water quality regulations. Subsequently, microcontrollers combined with algorithms [21–24] enables the implementation of data acquisition, analysis, prediction, management, and classification.

In the context of the smart city river monitoring project, a water quality monitoring system [25] based on the Internet of Things was proposed, which offered an affordable and efficient online monitoring scheme. To minimize the power consumption [26] of wireless sensor transmission, an energy-aware geographic routing algorithm was implemented. In addition, a microcontroller-based water contamination monitoring automation system [27] was introduced, which principally focused on monitoring indicators such as PH, temperature, turbidity, and dissolved oxygen. For other indicators, Turbidity was measured using a scattering method [28], while PH and conductivity were measured using an electrode method. IoT system [29] has been developed to analyze the Total Dissolved Solids (TDS), PH value, and turbidity. However, instead of real-time display and online acquisition, it utilizes an LED screen to display data. To address this issue, the IoT technology is utilized to monitor the data and summarize it for cloud-based data display [30]. To improve the efficiency of field monitoring network management, an improved algorithm [31] has been proposed. In addition, an algorithm [32] has been developed to address the issue of hardware-induced data synchronization problems, which can influence the monitoring process. A new precise technique [33] for software clock synchronization over a network of rigidly attached devices using gyroscope data was proposed.

References [32, 33] principally concentrate on ensuring the real-time transmission of data without analyzing the data monitored in sensor networks. To efficiently analyze data, the application of IOT and big data analysis technology [34] is introduced. A water quality index and classification system [35] are also developed to facilitate data analysis. Using data mining and the Internet of Things [36], Dang T, and their colleagues developed and implemented an online water quality monitoring system that serves as a tool for water resource management and environmental governance. With a focus on rivers, lakes, and other water bodies, literature [37] explored the recent advancements in information and communications technology and on-site sensor technologies for monitoring water quality. Furthermore, many scholars have also focused on analyzing outlier data in water quality and providing pollution early warning. To detect water quality changes, the indicator data is collected to construct an urban water supply system risk analysis framework [38], which solves the problems of monitoring water quality. An outlier detection clustering method [39] based on k-nearest neighbor is proposed, and its effectiveness has been validated using actual data sets. Likewise, the k-nearest neighbor distance [40] is used to represent the outlier score, and the upper threshold for detecting outliers is calculated. Several methods have been proposed to address the problem of outliers in water quality monitoring systems which can hinder information mining. One such method [41] utilizes the change-point grouping algorithm and the quartile algorithm to effectively identify four types of outliers. Additionally, a water quality monitoring system (WQMS) and water quality analysis algorithm [42] have been designed and the feasibility of the design scheme has been verified. Furthermore, a support vector algorithm [43] has been employed to con-struct a water quality model for reasonable analysis and prediction based on wireless monitoring. To attain water quality outlier data analysis and pollution early warning, an ontology modeling and rule generation method [44] has been proposed. While several applicable methods [34–44] have been designed for water quality data analysis, integrating these methods into an online analysis system can yield enhanced analysis results.

The present analysis reveals that online monitoring of water quality indicators and data analysis are two components of water quality monitoring. This study selects a low-power water quality monitoring sensor based on a comprehensive analysis of relevant literature and national standards for water quality indicator measurement to capture water quality data and conduct subsequent analysis and research. Indicators of temperature, pH, turbidity, conductivity, and total dissolved solids (TDS) are specifically chosen for water quality indicator monitoring. To achieve the objectives of rapid online measurement, data visualization, historical data management, and outlier data analysis, a water quality index monitoring and data analysis system is devised and developed for the Li River.

## Methods

The study does not involve activities that require specific permits, such as working with endangered species or in protected areas. In accordance with local regulations and guidelines, no permits are required for this study.

### Overall system composition

**Fig 1:**
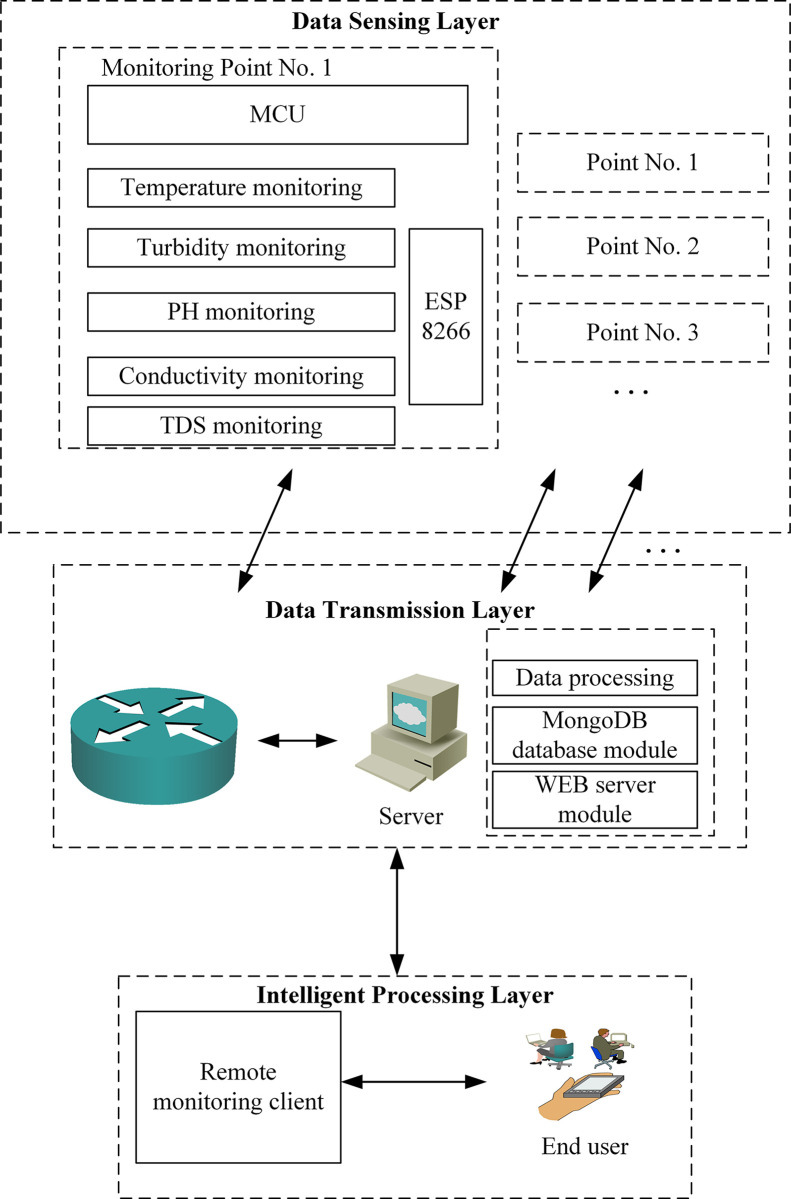
Overall structure design of the system.

The system is comprised of Data sensing layer, Data transmission layer and Intelligent processing layer. It has the functions of real-time monitoring, wireless data transmission, historical data management, data visualization and outlier data analysis. The overall design diagram is shown in Fig 1.

The Data sensing layer takes the microprocessor [45] as the core and contains various sensor modules and signal condition circuits. According to the type of water quality indicators to be collected, the PH signal condition circuit and PH electrode are selected to measure the PH; Select TS-300B turbidity module and conduct turbidity measurement; Select TDS sensor module for TDS measurement; Select conductivity electrode module for Conductivity measurement; Temperature has certain influence on the measurement of PH, Turbidity, Conductivity and TDS. To realize temperature compensation, the DS18B20 module is used for temperature measurement.

The Data transmission layer includes communication base station [46], core network, etc. Its primary objective is to transmit water quality data wirelessly to the server for subsequent processing. We have elaborated on the data transmission protocols employed in our system, including the utilization of USART and WebSocket protocols to facilitate seamless and efficient communication between different layers. Specifically, data transfer between the sensor and the STM32 is realized using the USART Protocol, and data transfer between the server side and the front end is realized using the WebSocket Protocol. The server-side software is developed using MongoDB database and Node.js technology.

The Intelligent processing layer is principally comprised of sensor real-time data visualization, historical data management, outlier data analysis, remote warning, and terminal software operation. Users record sewage data measured in accordance with national standard methods and manage, analyze, and visualize both real-time and national standard data. This layer enables efficient processing of data through its comprehensive functionality. our system leverages Node.js for server-side implementation and ECharts for visualizing data, ensuring robust and real-time data analysis.

A more detailed explanation of the communication protocols from the data sensing layer to the data transmission layer and intelligent processing layer are as follows.

#### USART protocol

The Universal Synchronous Asynchronous Receiver Transmitter (USART) is a serial communication protocol employed for serial data transmission in digital systems. It offers the flexibility of operating in either synchronous or asynchronous mode, making it versatile and widely utilized in embedded systems and communication devices, facilitating the connection of microcontrollers, sensors, storage devices, and other peripherals. In this paper, we utilize it for communication between sensor data and microcontrollers. The key features include:

**1. Synchronous and asynchronous modes:** The USART offers the flexibility to switch between synchronous and asynchronous modes. In synchronous mode, external clock signals synchronize communication devices, while asynchronous mode utilizes start and stop bits for data frame synchronization.

**2. Full-Duplex communication:** The USART supports full-duplex communication, enabling the device to send and receive data simultaneously without the need to switch modes.

**3. Baud Rate setting:** The baud rate is a crucial parameter that determines the data transmission rate in USART communication. With USART, users can change the baud rate to meet various application requirements.

**4. Data Frame format:** USART supports a variety of data frame forms, allowing the user to specify the number of data bits, parity bits, and stop bits.

The main operating principles are outlined as follows:

**1. Transmitter:** Depending on the selected mode, the transmitter loads data into the USART transmit buffer, appends the start bit, data bits, check bit, and stop bit, and then transmits the data sequentially, bit by bit.

**2. Receiver:** The receiver monitors the USART receive buffer, reads the received bits, and reconstructs the original data following the same frame format. The receiving end may perform checksums to ensure data integrity.

**3. Clock:** The timing of data transfer in synchronous mode is controlled by an external clock. A baud rate generator is used in asynchronous mode to facilitate coordination between the transmitter and receiver.

#### Transmission control protocol

The Transmission Control Protocol (TCP) is a reliable, connection-oriented, and byte-stream communication protocol at the transport layer. It establishes a connection using a three-way handshake, as outlined in the specific process below.

1. The client chooses an initial sequence number and sends a TCP segment with the "SYN" (synchronize) flag set. The client is now in the "SYN_SENT" state.
2. The server replies with a TCP segment that has its selected beginning sequence number, the "ACK" (acknowledge) and "SYN" flags set, after receiving the "SYN" from the client. The server is now in the "SYN_RECV" state.
3. The client transmits an "ACK" segment, and the TCP connection is formed after receiving the "SYN" and "ACK" segments from the server. Data transfer is now possible since both parties have reached the "ESTABLISHED" state.

This study utilizes the TCP protocol to transmit data to the server, as outlined below.

1. A TCP server program is developed on the server side, and port 4001 is opened to listen for connections from the client. A partial screenshot of the code is shown below, and the complete code can be found in the "server.js" file within the "bin" folder. The Node.js Net module provides tools for network communication.
2. For network connections and server access, the ESP8266 module is used in hardware module network programming. First, the network account and connection password are configured in the hardware code. Second, the IP address and connection port of the server are mentioned. The ESP8266 module transmits data using the integrated TCP protocol and the STA (Station) operating mode.
3. The server keeps device information and real-time water quality data in the database upon a client connection. TCP communication now continues normally.

#### WebSocket protocol

WebSocket is a network communication protocol built upon TCP, designed to address a limitation of the HTTP protocol: the inability of the server to proactively send messages to the client. Key features of the WebSocket protocol include:

**1. Bidirectional communication:** WebSocket enables the establishment of a persistent connection between the client and server, facilitating bidirectional real-time communication. In contrast to the request-response model of the HTTP protocol, WebSocket significantly reduces latency and improves efficiency.

**2. Full-Duplex communication:** WebSocket facilitates full-duplex communication, enabling the client and server to send data simultaneously without having to wait for a response. This bidirectional flow of data simplifies the development of real-time applications, such as online chat and collaborative platforms.

**3. Reduced data transmission overhead:** WebSocket transmits data in frames, minimizing unnecessary header overhead compared to the HTTP protocol. This reduction in data transmission burden improves overall efficiency and responsiveness.

**4. Handshaking process:** The WebSocket protocol initiates a handshaking process during connection establishment. The process starts with a protocol upgrade request using the HTTP/HTTPS protocol, allowing subsequent communication via a TCP connection.

The WebSocket protocol is used in the construction of this system for two primary purposes:

**1. Real-time data transmission from server to web page:** A web page receives data from the server, which continuously listens for real-time data from hardware devices. In this process, the "ws" module in Node.js is used to implement the WebSocket capabilities.

**2. Visualization of server-sent data on a web page:** The web page actively receives and displays data sent in real time from the server. The WebSocket object, generated within the JavaScript code of the web page, is essential for implementing this feature in this case.

#### AJAX technology

Asynchronous JavaScript and XML(AJAX) Technology is a set of web development techniques that enable web pages to be updated asynchronously by exchanging small amounts of data with the server behind the scenes. Web applications that utilize it can quickly display incremental changes to the user interface without reloading or refreshing the page. This improves the program’s responsiveness to user input.

Key features of Ajax technology include:

**1. Asynchronous Operation:** Ajax operates asynchronously, allowing it to transmit and receive data in the background from a web server without impacting the appearance and behavior of the website.

**2. XMLHttpRequest Object:** The foundation of Ajax is the XMLHttpRequest object. It offers the ability to handle server responses asynchronously and to send HTTP requests to the server.

**3. DOM Manipulation:** With Ajax, programmers can dynamically modify the Document Object Model (DOM), updating specific sections of a webpage without requiring a complete page reload.

#### Node.js technology

The server-side program is implemented using Node.js, which is an open-source, cross-platform JavaScript runtime environment. Renowned for its versatility, Node.js is a popular choice for a wide range of projects, including real-time and chat applications. It can be used to develop tools, desktop applications, and backends for Internet of Things (IoT) devices. Node.js is incredibly efficient because it runs the V8 JavaScript engine, which is the foundation of Google Chrome, outside of the web browser. In comparison to Java, Python, and other programming languages, using Node.js for small and medium-sized web applications can improve efficiency. The Node.js structure comprises three main levels:

**1. Node.js standard library:** This top layer consists of an array of JavaScript code, providing developers with application programming interfaces (APIs). It provides advanced features for managing HTTP requests, developing web applications, handling files on the system, and more.

**2. Node bindings:** Node Bindings is the intermediate layer that acts as a bridge between JavaScript (JS) and lower-level languages such as C/C++. To enable communication between JavaScript and the underlying system or external C/C++ libraries, this layer is essential. It serves as a conduit for JavaScript native code execution.

**3. Foundational components:** C/C++ is used to implement Node.js’s bottom layer. Along with other essential features, it includes the V8 JavaScript engine for runs JavaScript code, and libuv, a cross-platform asynchronous I/O library, among other components. This layer serves as the Node.js’s fundamental building block, providing the infrastructure needed to effectively manage low-level processes.

The strength and adaptability of Node.js are enhanced by its tiered architecture, which enables programmers to create scalable and efficient systems by utilizing both lower-level C/C++ capabilities and higher-level JavaScript APIs.

#### MongoDB

Developed primarily in C++, MongoDB is a notable example of a non-relational, document-based database. Unlike conventional relational databases, MongoDB allows for the dynamic addition, deletion, and alteration of fields as needed because it places less restrictions on fields. Because of its adaptability, it can handle large amounts of data that are collected in real time from multiple sources. This enables its use in a variety of scenarios, such as real-time analytics, log collection and storage, and Internet of Things (IoT) applications. MongoDB is used as the data storage solution in the development of this system, while "Studio 3T" is the tool used for efficient database management.

#### ECharts technology

ECharts (Enterprise Charts) is a JavaScript-based open-source visualization chart library developed by Baidu. It provides a diverse range of chart formats, such as line graphs, bar charts, pie charts, and scatter plots for data visualization. Developers can easily download the requisite ECharts JavaScript file from the official website and integrate it into the system. In this system, it is mainly used for real-time data visualization.

Here is an introduction to the key features of ECharts:

**1. Responsive design:** ECharts can automatically adjust the size and style of charts in response to changes in window size, thanks to its responsive design features. This ensures compatibility with various screen sizes for improved user experience.

**2. Mobile support:** ECharts is optimized for mobile devices, allowing for chart interaction on smartphones and tablets. Its mobile support improves the accessibility of charts on various devices.

**3. Excellent compatibility:** ECharts is compatible with popular web browsers, such as Chrome, Firefox, Safari, Edge, and others. This interoperability ensures a reliable and consistent charting experience across multiple platforms.

### System hardware design

**Fig 2:**
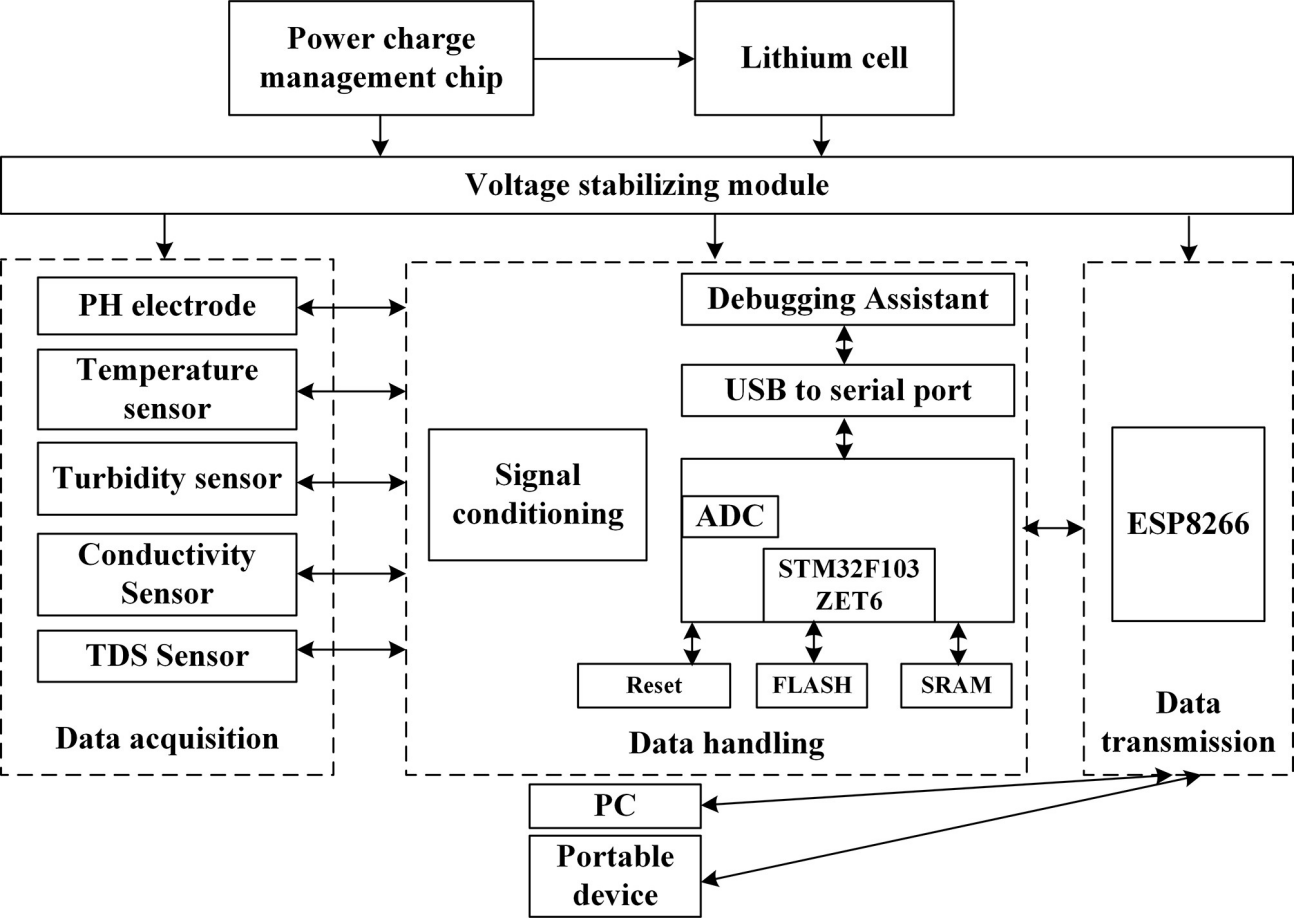
Overall hardware design of the system.

The water quality measurement module principally includes PH value measurement module, Temperature measurement module, Turbidity measurement module, Conductivity measurement module and TDS measurement module. The overall design of the hard-ware module is shown in Fig 2. Additionally, sensor-related parameters and calibration methods have been introduced in each module.

The MCU [47] obtains the sensor data, and processes it through the signal condition module. Subsequently, the multiple streams of sensor data are integrated and transmitted to a remote server via the WIFI module for efficient processing. It is helpful for the efficient management and analysis of multiple sensor data streams.

#### PH measurement module

According to the national standard HJ1147-2020, the glass electrode method is man-dated for determining the PH of surface water. Drawing on the Nernst equation, a module has been developed to enable online PH detection. The E-201-CF PH composite electrode serves as the crucial component of the PH meter. Given that temperature information is requisite for PH measurements, a DS18B20 temperature sensor featuring waterproof packaging and strong adaptability is utilized. The relevant parameters of the sensor module are presented in Table 2 below and are found to satisfy the essential measurement requirements for surface water.

**Table 2:** Detailed explanation of pH module.

Parameter	Value	Parameter	Value
Operating voltage	5.00V	Response time	≤1min
Measurement range	0–14	PH sensor interface	BNC
Operating temperature	0–60°C	Temperature sensor interface	XH2.54

Before using the pH module, pH calibration must be performed to account for the variations in pH electrodes and possible resistance faults in potentiometers. Please follow the specific steps outlined below:

1. Connect the pH sensor module to the electrode and supply the module with 5 volts to improve accuracy.
2. Carefully remove the electrode protection cap, ensuring that the bulb protection solution does not spill. The electrode should be immersed in a pH 6.86 standard buffer solution. To achieve a PO port voltage of about 2.52V, adjust the potentiometer knob. Set the PO port voltage to approximately 1.7V if the AD conversion acquisition voltage range is 0~3.3V.
3. Immerse the electrode in a pH 4.00 standard buffer solution. To achieve a PO port voltage of about 3.00V, adjust the potentiometer knob. Set the PO port voltage to 2.2V if the AD conversion voltage range is 0~3.3V.
4. Immerse the electrode into a pH 9.18 standard buffer solution. To get the PO port voltage to be approximately 2.12V, adjust the potentiometer knob. Set the PO port voltage to approximately 1.3V if the AD conversion acquisition voltage range is 0~3.3V. The pH module has now been calibrated.

#### Turbidity measurement module

The national standard HJ 1075–2019 prescribes a method for determining the turbidity of water, which is applicable to the determination of groundwater and surface water. At present, two common methods are transmission methods and scattering methods. Most turbidity meters are developed based on these two methods. The scattering method is particularly applicable for measuring low turbidity solutions. When a constant light source is transmitted through the solution, a linear relationship between the particle concentration and total scattered light intensity can be observed. In this paper, a model turbidity sensor is utilized and equipped with a signal condition module. The parameters of this module are summarized in Table 3.

**Table 3:** Turbidity sensor module parameters.

Parameter	Value	Parameter	Value
Operating voltage	5.00V DC	Response time	<500ms
Operating current	40mA(MAX)	Analog output	0~4.5V;
Measurement range	0~1000NTU	Digital output	high/low level signal
Operating temperature	-20 ~90°C	Sensor interface	XH2.54

As shown in Table 3 above, the sensor module, developed utilizing the scattering method, has demonstrated its suitability for measuring turbidity in surface water, meeting the essential criteria for such measurements.

To account for individual variances in turbidity sensors, their susceptibility to ambient light, and the need for temperature compensation, calibration is essential to ensure more accurate turbidity results. The method for calibrating the turbidity module is outlined below:

1. Connect the turbidity sensor module. For calibration, use pure or distilled water that is close to 0 NTU, or use a standard solution with 0 NTU.
2. Note the temperature value of the calibration solution as *T*_*t*_ (decreasing mistakes due to light intensity). Measure the sensor module’s output voltage as *U*_*t*_ simultaneously.
3. To determine the voltage differential *ΔU* brought on by the temperature fluctuation, enter the temperature value *T*_*t*_ into the correction formula.
$$\Delta U=-0.0192\times (T_{t}-25)$$ (1)
4. To calculate *U*_25°C_, subtract the voltage value *U*_*t*_ from the voltage difference Δ*U*. To find the value of *K*, enter *U*_25°C_ into the standard curve formula.
$$U_{25{}^{\circ}C}=U_{t}-\Delta U$$ (2)
$$K=865.68\times U_{25{}^{\circ}C}$$ (3)
5. Modify the standard curve formula by adding the determined *K* to the formula.
TU=−865.68×U+K (4)

#### Conductivity measurement module

Conductivity is a critical parameter for assessing water quality, which is closely related to salinity and total hardness indicators. Therefore, these two indicators can be deduced from conductivity, which helps to reduce measurement indicators and improve measurement efficiency. The national standard JJG 376–2007 prescribes methods for measuring conductivity, and the parameters are summarized in Table 4 below.

**Table 4:** Detailed explanation of conductivity indicators.

Parameter	Value	Parameter	Value
Operating voltage	5.00V	Support measurement range	0~20mS/cm
Operating temperature	0–40°C	Recommended measurement range	1~15mS/cm
Measurement accuracy	±5%F.S.	Electrode life	>0.5 year
Conductivity cell constant	1.0	Cable length	100cm

Due to the high price of professional conductivity measuring instruments, which is not conducive to secondary development, conductivity condition modules and corresponding electrode secondary development are selected. As shown in Table 4 above, the measuring range and service life of this electrode satisfy the requirements of measurement.

The conductivity module’s calibration process is as follows. Electrodes that are used for the first time or for a prolonged period must be calibrated for accuracy. A 2-point calibration method is applied, using conductivity standards of 1413 uS/cm and 12.88 mS/cm. The following is a list of the precise operating steps:

1. Connect the sensor module and the electrode to the power supply. Immerse the electrode into the standard solution with a conductivity of 1413uS/cm. Measure the output voltage from the AO port and record it as *V*_*t*_. Record the current temperature of the solution as *T*_*t*_. Substitute these values into the Formula (5).

$$kValue\_Low=\frac{164\times 1.413\times (1.0+0.0185\times (T_{t}-25.0))}{V_{t}}$$ (5)

2. Immerse the conductivity electrode into the standard solution with a conductivity of 12.88 mS/cm. Obtain the new AO port voltage and temperature values and enter them into the following Formula (6).

$$kValue\_High=\frac{164\times 12.88\times (1.0+0.0185\times (T_{t}-25.0))}{V_{t}}$$ (6)

3. Modify the K value by correcting the computed *kValue*_*High* and *kValue*_*Low* values in the program. To finish the conductivity module calibration, compile and burn the amended program.

#### TDS measurement module

The national standard GB5749-2022 prescribes the limits of TDS concentration. Furthermore, there is a distinct correlation between the TDS indicator and the conductivity indicator, which helps to partially cross-validate the results of both measures. While the conventional TDS detection pen is user-friendly, it is not equipped for online measurement, data storage, and analysis. Therefore, the development of a secondary TDS circuit module and TDS probe has been undertaken. The parameters are summarized in Table 5.

**Table 5:** Detailed explanation of TDS.

Parameter	Value	Parameter	Value
Measurement range	0~1000ppm	Module size	42mm×31.2mm
Measurement accuracy	±5%F.S. (25°C)	Probe interface	2Pin XH-2.54
Operating current	3~6mA	Cable length	58CM
Operating voltage	3.3~5.0V	Temperature of solution	≤70°C
Output signal range	0~2.3V	Insulation resistance	≥50MΩ
Temperature sensor interface	3Pin XH-2.54		

Variations in Total Dissolved Solids (TDS) probes or the absence of temperature compensation can lead to measurement errors. Calibration must be performed to guarantee higher precision in TDS measurements. Furthermore, it is recommended to connect a temperature sensor and apply temperature compensation to improve measurement accuracy. The specific operational steps are outlined below.

1. Connect the TDS probe and temperature sensor. Then, using a TDS pen or the standard TDS solution, find the solution’s TDS value, which is represented by the symbol *TDS*_*s*_ (Total Dissolved Solids Standard Value).
2. Power up the TDS sensor module, immerse the temperature sensor and TDS probe in the solution, and record the output voltage as *V*_*t*_ from the AO port. Concurrently, note the temperature of the solution as *T*_*t*_. To determine the corrected output voltage, *V*_*c*_, use the measured values of *V*_*t*_ and *T*_*t*_ in Eqs (7) and (8) for the temperature adjustment coefficient. After that, enter *V*_*c*_ into Eq (9) of the TDS standard curve to get the final corrected *TDS*_*t*_ (Total Dissolved Solids Test Value).

$$T_{c}=1+0.02\times (T_{t}-25)$$ (7)

$$V_{c}=T_{c}\times V_{t}$$ (8)

$$TDS_{t}=(66.71\times V_{c}^{3}-127.93\times V_{c}^{2}+428.7\times V_{c})$$ (9)

3. Using the following Equation, find the K value, assuming that *TDS*_*s*_ is 90 ppm and *TDS*_*t*_ is 100 ppm. *K* is estimated to be 0.9.

K=TDSsTDSt (10)

4. Update the *K* value in the program, making sure that the kValue is changed to correspond with the determined *K* value.

#### Temperature measurement module

The DS18B20 is a widely used digital temperature sensor. It produces a digital signal and is known for its compact size, strong anti-interference capability, and high accuracy. Providing digital output helps to reduce errors in data transmission. Furthermore, the sensor undergoes pre-calibration before leaving the factory, offering convenience to users by eliminating the need for additional calibration steps during use. This feature ensures that the sensor meets the practical requirements of the system. Its accuracy within the range of -10°C to +85°C is ± 0.5°C. The parameters are summarized in Table 6.

**Table 6:** Detailed explanation of temperature sensor.

Parameter	Value	Parameter	Value
Operating voltage	3.5~5.00V	Protocol	1-Wire
Resolution	9~12bits	Support measurement range	−55~125°C
Measurement accuracy	±0.5°C	Hardware overhead	low

### System software overall design

**Fig 3:**
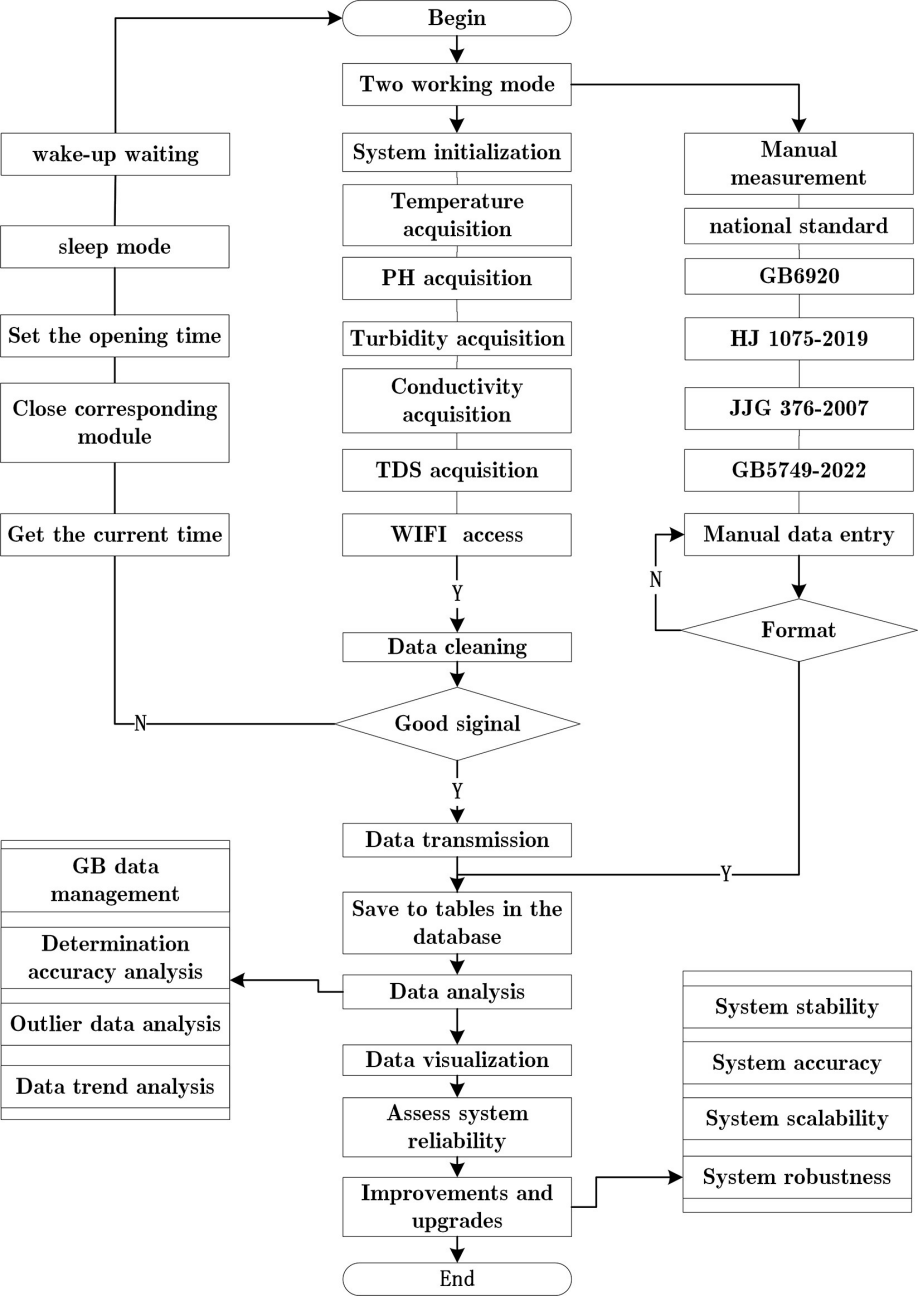
System software operation flow chart.

Fig 3 depicts the flow charts of the software of the system. The software aspect of the system offers two distinct operating modes to facilitate data comparison experiments. The first mode utilizes the system devised in this paper to conduct experiments, while the second mode conducts experiments based on the national measurement standards.

As illustrated in Fig 3 above, applicable code has been composed to capture the five indicators. Due to limitations in the processing capacity of the microprocessor, the multiple streams of sensor data are concatenated and transmitted to the server as a single entity. The server carries out data cleaning and saves legal data to facilitate data analysis and visualization. Based on the issues identified in the experiment, the system can be upgraded to further optimize its stability, accuracy, and scalability.

In Fig 3, more detailed information is provided about data collection and the corresponding national standards. Specifically, it includes the standard method for measuring pH indicators in GB6920 and the standard method for measuring turbidity indicators in HJ1075-2019. JJG 376–2007 corresponds to the standard method for measuring conductivity indicators. GB5749-2022 corresponds to the standard method for measuring Total Dissolved Solids (TDS) indicators. Based on these national standards, comparative experiments are conducted, and detailed information can be found in the Comparative analysis of two measurement methods section.

Fig 3 also introduces the primary functions of the intelligent processing layer, such as data analysis and visualization, system improvement, and upgrading, etc. It starts with hardware data collection and splicing, followed by data transmission, and finally data analysis and improvement, which is presented in the form of a flowchart to provide additional details.

### Sewage indicator monitoring module

**Fig 4:**
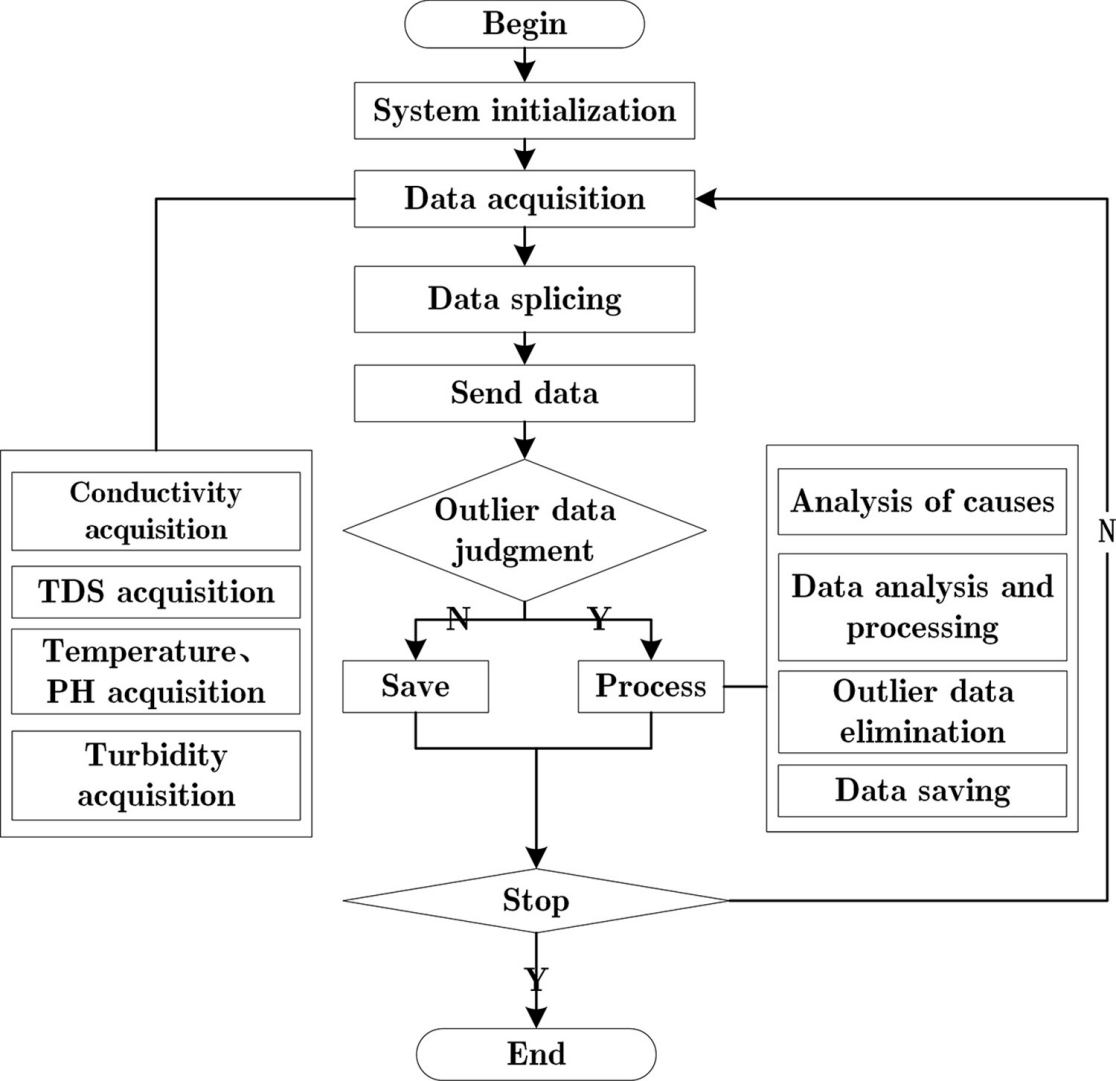
Flow chart of sewage indicator monitoring module.

The flowchart of the sewage indicator monitoring module is presented in Fig 4 below. The microprocessor acquires data from the sensor and transmits it to the WIFI module through the serial port. The module, in turn, forwards the data to the server. Due to the limited processing capacity of the microprocessor, an outlier data detection program has been implemented and executed on the server. In cases where interrupt operation is not utilized by the system, the process is repeated.

During data collection, water quality sensors are prone to generating errors, necessitating the identification and rectification of outliers data. Anomalous data can be categorized as syntax errors and numerical outliers. Eliminating data with syntax errors is a straightforward task. However, for data featuring numerical outliers, this research utilizes the quartile method [48] to discern them. The specific process is as follows:

First, we acquire the monitoring sequence for a period and sort it in ascending order, let *X* = [*x*_1_,*x*_2_,…,*x*_*n*_] be a sample of size n. It is divided into four parts on average, and each part is 25%. *q*_1_, *q*_2_ and *q*_3_ respectively represent the lower quartile, the median and the upper quartile.


$$q_{2}=\{\begin{array}{c} x_{(n+1)/2}\\ \frac{x_{n/2}+x_{n+2/2}}{2} \end{array}\;\begin{array}{l} n=2k+1;k=0,1,2,\ldots \\ n=2k;k=0,1,2,\ldots \end{array}$$ (11)

When *n* = 4*k*+3(*k* = 1,2,…), The calculation formula is as follows.


$$\{\begin{array}{c} q_{1}=\frac{3}{4}x_{k+1}+\frac{1}{4}x_{k+2}\\ q_{3}=\frac{1}{4}x_{3k+2}+\frac{3}{4}x_{3k+3} \end{array}$$ (12)

When *n* = 4*k*+1(*k* = 1,2,…), The calculation formula is as follows.


$$\{\begin{array}{c} q_{1}=\frac{1}{4}x_{k}+\frac{3}{4}x_{k+1}\\ q_{3}=\frac{3}{4}x_{3k+1}+\frac{1}{4}x_{3k+2} \end{array}$$ (13)

The definition of Interquartile interval is as follows.


$$\left[F_{1},F_{2}]=[q_{1}-1.5IQR,q_{3}+1.5IQR\right]$$ (14)

We utilize the quartile method to identify outliers present in the sensor data, followed by the utilization of seven distinct methods to rectify these outliers.

### Data analysis module

The system supports multi-point monitoring, enabling for the selection of sensors located in various positions for data analysis. The data analysis module primarily includes national standard data management, measurement precision analysis, outlier data analysis and data trend analysis.

**Description of the dataset.** Historical data analysis and outlier data analysis are the two primary components of the data analysis module. An overview of the datasets utilized for data analysis is provided below.

*1*. *Historical data analysis dataset*. After successfully developing the system, experiments were conducted to measure water quality, and the collected data were stored in the MongoDB database, namely in the "auto_msg" collection. Data collection started in early October 2022 and continued until December 2023, with the bulk of the data being collected between October and December 2022. With over 100,000 entries in the dataset, the measurement took place over the course of around a year and a half. The detailed introduction of the historical data analysis dataset is summarized in Table 7.

**Table 7:** The detailed introduction of the historical data analysis dataset.

Attribute	Details
Dataset size	Over 100,000 records
Measurement Start Time	October 2022
Measurement End Time	December 2022
Data Density	Relatively high-density data was collected between September and December 2022.
Storage Location	In mongodb database, ’auto_msg’ collection
Time Span	Approximately one and a half years (14 months)

To facilitate system debugging, the sample interval was set to 2 seconds during the measurement phase. The interval can be adjusted as needed in later steps. The structure of the "auto_msg" collection is summarized in Table 8 below. For easy import/export in the MongoDB database, the data will be exported in JSON format and uploaded with the paper.

**Table 8:** The structure of the collection.

Field Name	Data Type	Description
_id	ObjectId	Unique identifier in the database
devId	String	Device identifier
date	String	Recording date
year	Integer	Recording year
season	Integer	Recording season
month	Integer	Recording month
day	Integer	Recording day
currentTime	String	Recording time
turbidity	Float	Turbidity (NTU)
temperature	Integer	Temperature (in Celsius)
ph	Float	PH (-)
tds	Float	Total Dissolved Solids (TDS)
ec	Float	Conductivity (EC)

*2*. *Dataset for outlier analysis*. As illustrated in Table 9 below. When analyzing outlier data, it is required to extract data from the same water sample, as multiple water samples were collected at different times. To accomplish this, data is extracted from five different time periods, designated Data1 through Data5, each associated with a single water sample. We will conduct outlier data analysis on the items in these datasets, which are subsets of the historical data analysis dataset. To facilitate reproducibility, the data from these five periods will be exported in CSV format and uploaded with the paper.

**Table 9:** The detailed introduction of the outlier data analysis dataset.

Data No	Data date	Quantity of data
1	2022/10/13	777
2	2022/11/7	1828
3	2022/11/14	1948
4	2022/11/21	2769
5	2022/12/9	3108

#### Data analysis requirements

For historical data analysis, the primary requirements are as follows:

**1. Customizable analysis conditions:** Provide users with an intuitive query interface that includes options for selecting date ranges and devices. This improves the overall user experience by simplifying the process of setting analytic conditions. Users should be able to specify the range of data they want to retrieve, for example, the last six months or the last year. Additionally, users have the option to select specific devices from the database. After submitting the query, the system should mark the date with the most data in the line chart.

**2. Visual analysis results:** When a user submits a query and data exists, display the dates within the specified range as a line graph. If no data is found, the interface should display as empty.

**3. Providing reference:** Visual aids should enable users to quickly grasp the overall data pattern. The line graph, as a visual representation, facilitates the comprehension of averages, extremes, and other indicators of water quality derived from data analysis. This provides users with a quick reference to understand the current state of the water quality.

In addition, we select and analyze the data for outlier analysis based on the following criteria:

**1. Cross-Month data sampling:** To ensure a representative and diverse dataset for outlier analysis, data will be collected over multiple months.

**2. Targeted data sampling:** Using the turbidity index as an example, we will test different turbidity solutions (e.g., lower and moderate turbidity) to gather a diverse range of datasets. This will help us meet the requirement for specificity.

**3. Minimum data quantity for analysis:** Each dataset used for a single analysis must contain a minimum of 300 data points. The aim of this criterion is to facilitate a thorough assessment of the performance of various sensors.

#### Data analysis process

**Fig 5:**
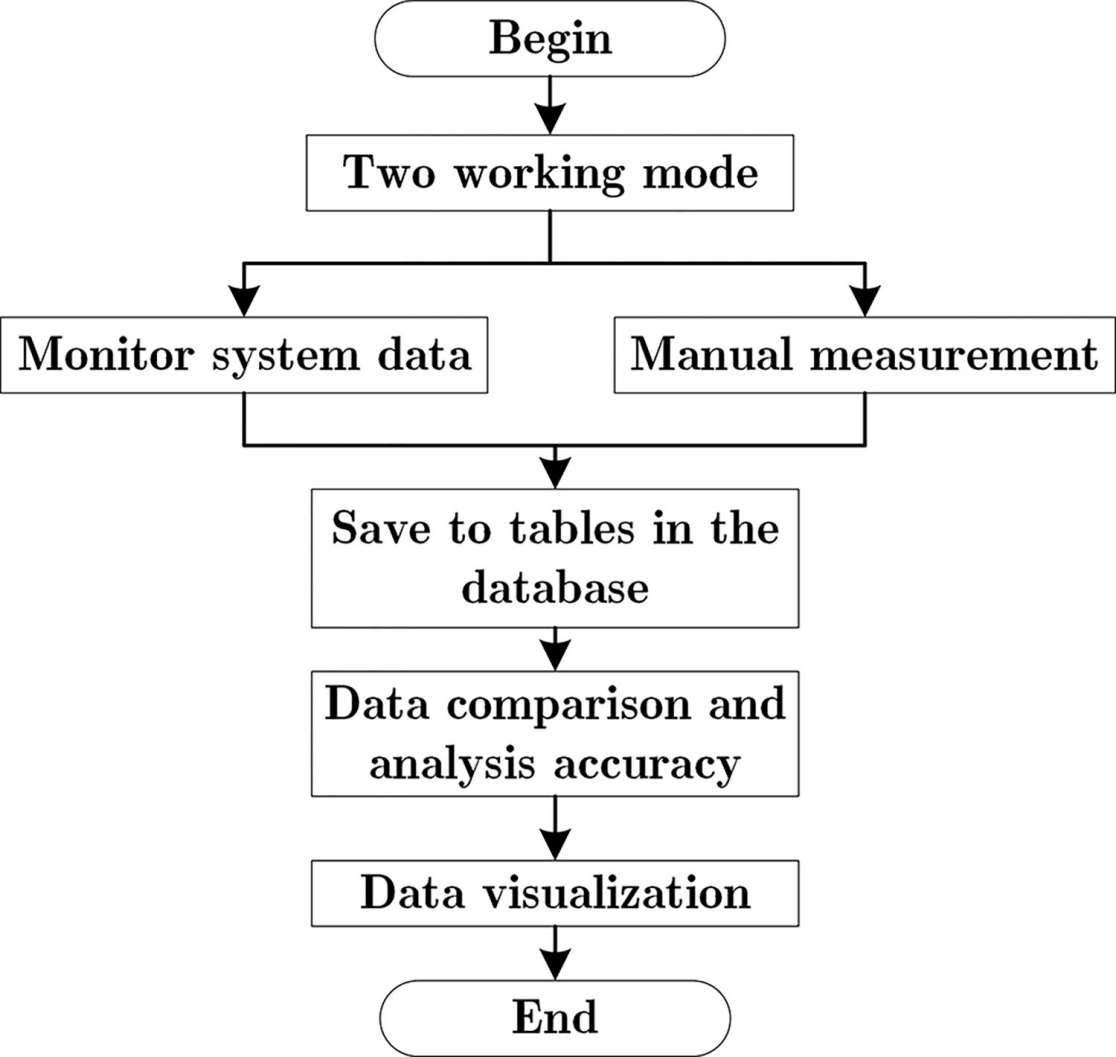
Flow chart of data precision analysis module.

The design flow chart of the data precision analysis module is shown in Fig 5. In the water quality determination, the two groups of data of comparative trial are stored in the database for comparison, analysis, and visualization.

**Fig 6:**
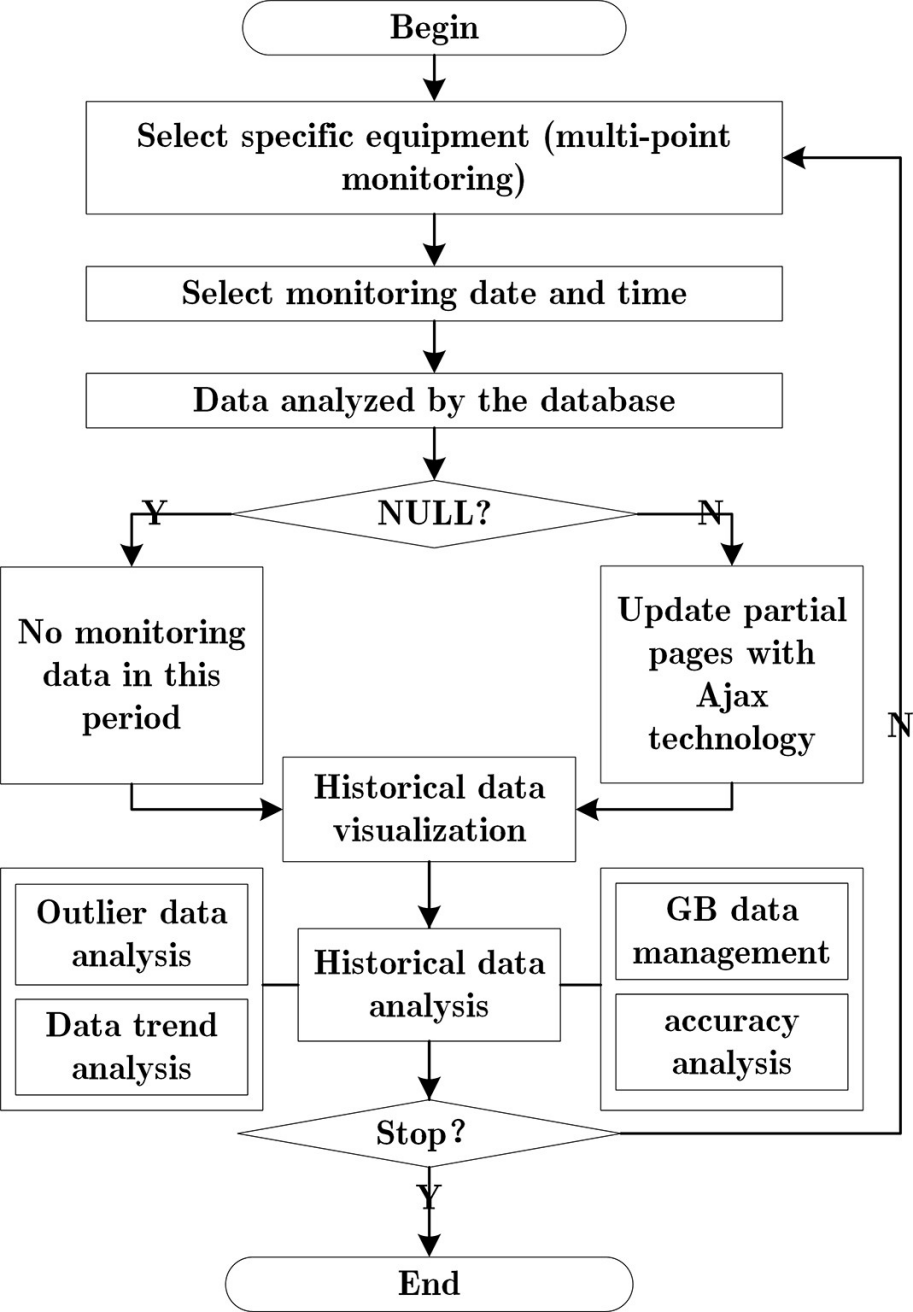
Design flow chart of historical data management.

The system also analyzes and manages historical data. It obtains the data distribution characteristics of PH, Turbidity and Temperature, Conductivity and TDS value at a given time. To better display the results, Asynchronous Javascript And XML (AJAX) and Echarts technology are primarily used to get the effect of updating and visualization without refreshing pages. The design flow chart is shown in Fig 6.

In the context of outlier data analysis and processing, the identification of outliers through the application of the quartile method is the initial phase. The subsequent step entails the correction of these outliers, for which a number of techniques are available, such as direct deletion, mean filling, mode filling, median filling, Lagrange interpolation, and regression classification prediction filling. The regression classification prediction filling method utilizes existing data for prediction training, which may result in a significant increase in response time for systems with stringent real-time requirements. Consequently, this study predominantly employs seven outlier rectification techniques, namely mode filling, mean filling, median filling, Lagrange interpolation filling, k-nearest neighbor filling, the use of the previous non-empty element filling, and direct deletion.

## Results and discussions

It primarily involves the following tests, including real-time data display function, historical data management function, outlier data analysis and comparative analysis of the two measurement methods.

### Real-time data display

**Fig 7:**
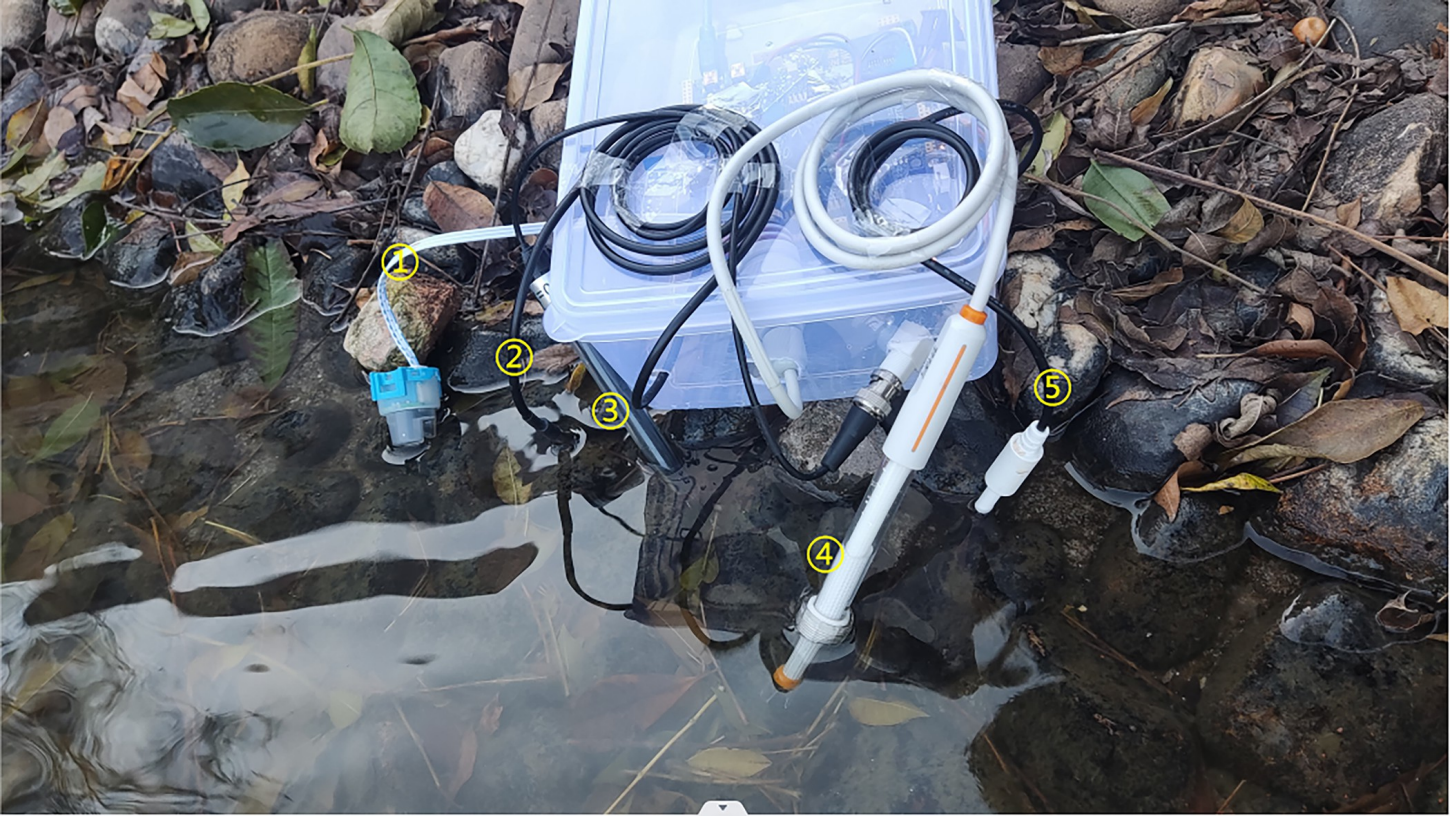
A test in an actual river environment.

The images taken in actual river environments are shown in Fig 7 below. Markers ①, ②, ③, ④, and ⑤ in the figure correspond to the turbidity sensor, temperature sensor, conductivity sensor, PH sensor, and TDS sensor, respectively.

**Fig 8:**
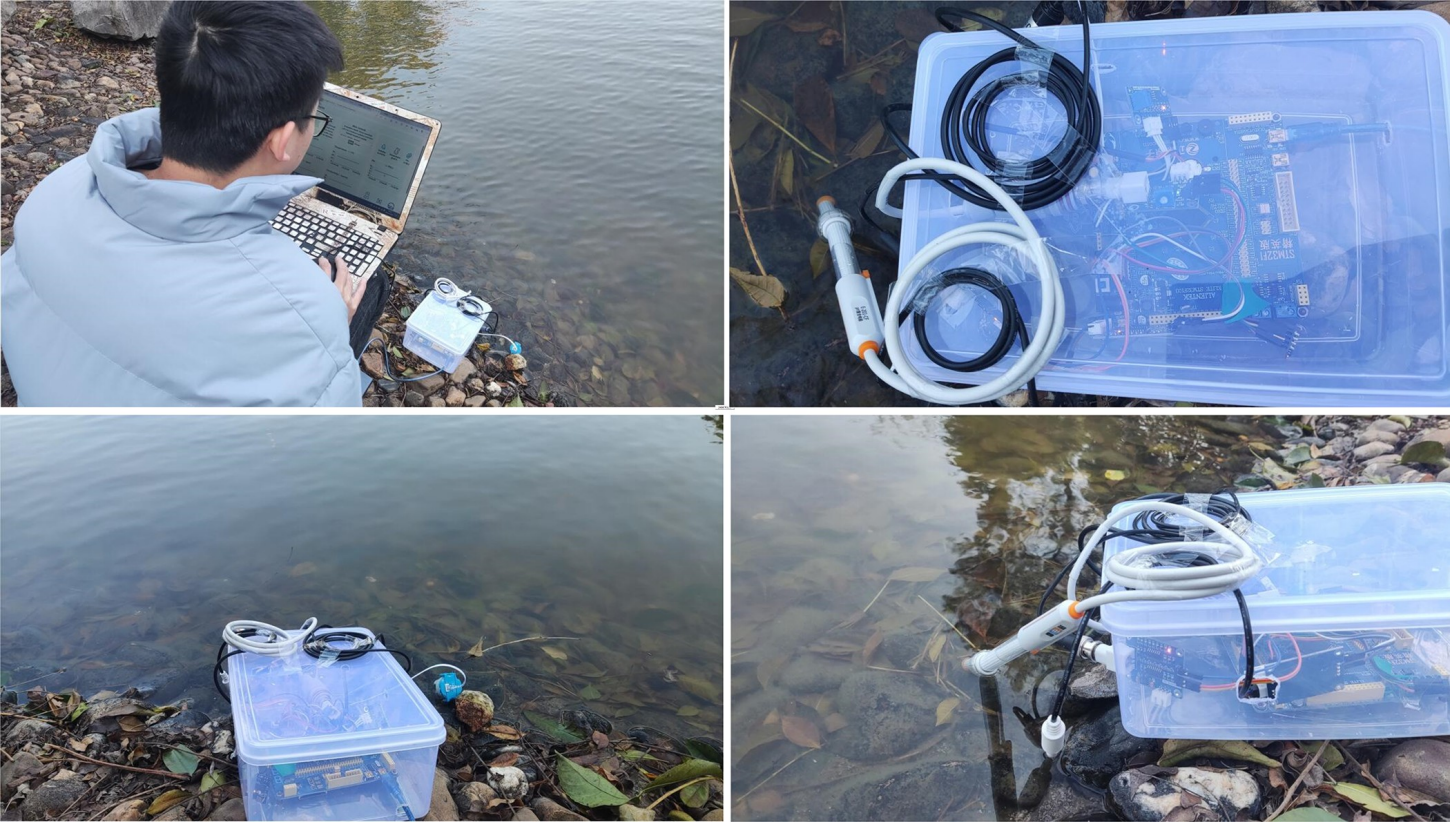
Other images in actual river environments. (A) Device debugging in a real environment, (B) The top view of hardware equipment, (C) The front view of hardware equipment, (D) The side view of hardware equipment.

To ensure reliability under various circumstances, we will also follow standard calibration methods in the case of changes to the measuring environment. Furthermore, other images measured in the field are shown in Fig 8.

As depicted in the Fig 8A above, we performed system and device debugging in an actual environment. In addition, Fig 8B–8D show the top view, front view, and side view of the device. the locations where we regularly sample the river on a map, as illustrated below.

**Fig 9:**
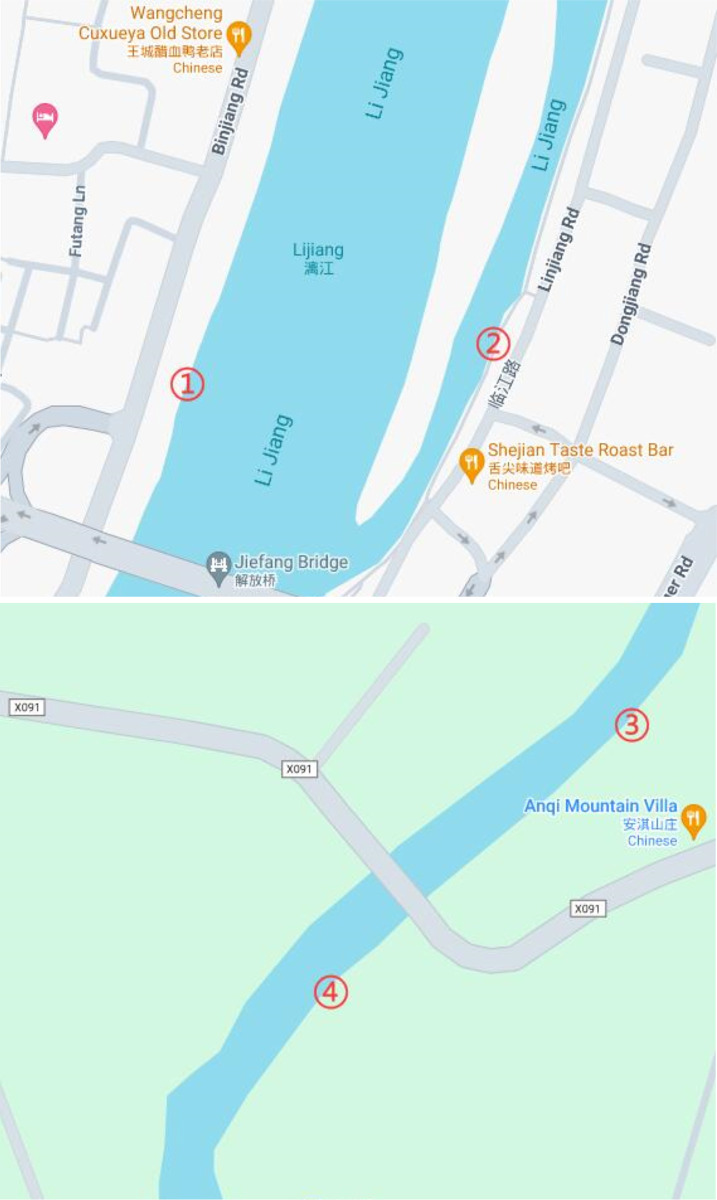
Location of water samples on the map. (A) Sampling locations along the Li River, (B) Sampling locations along the tributaries.

As shown in the Fig 9, we conducted sampling at four locations in the Li River and its tributaries in Guilin City. Each point is numbered, and its corresponding latitude and longitude are listed in the table below. Sampling Points 1 and 2 are situated in the mainstream of the Li River, while Sampling Points 3 and 4 correspond to one of the tributaries, the Xiangsi River. Since the TDS, conductivity, turbidity, temperature, and pH sensors are all connected to the same microcontroller, they measure different indicators of the same water body. Their testing locations are identical and can be referenced in the Table 10.

**Table 10:** Longitude and latitude of water sample position.

Location number	Latitude	Longitude
①	25.278470	110.302652
②	25.278693	110.305195
③	25.190415	110.342916
④	25.188348	110.340352

**Fig 10:**
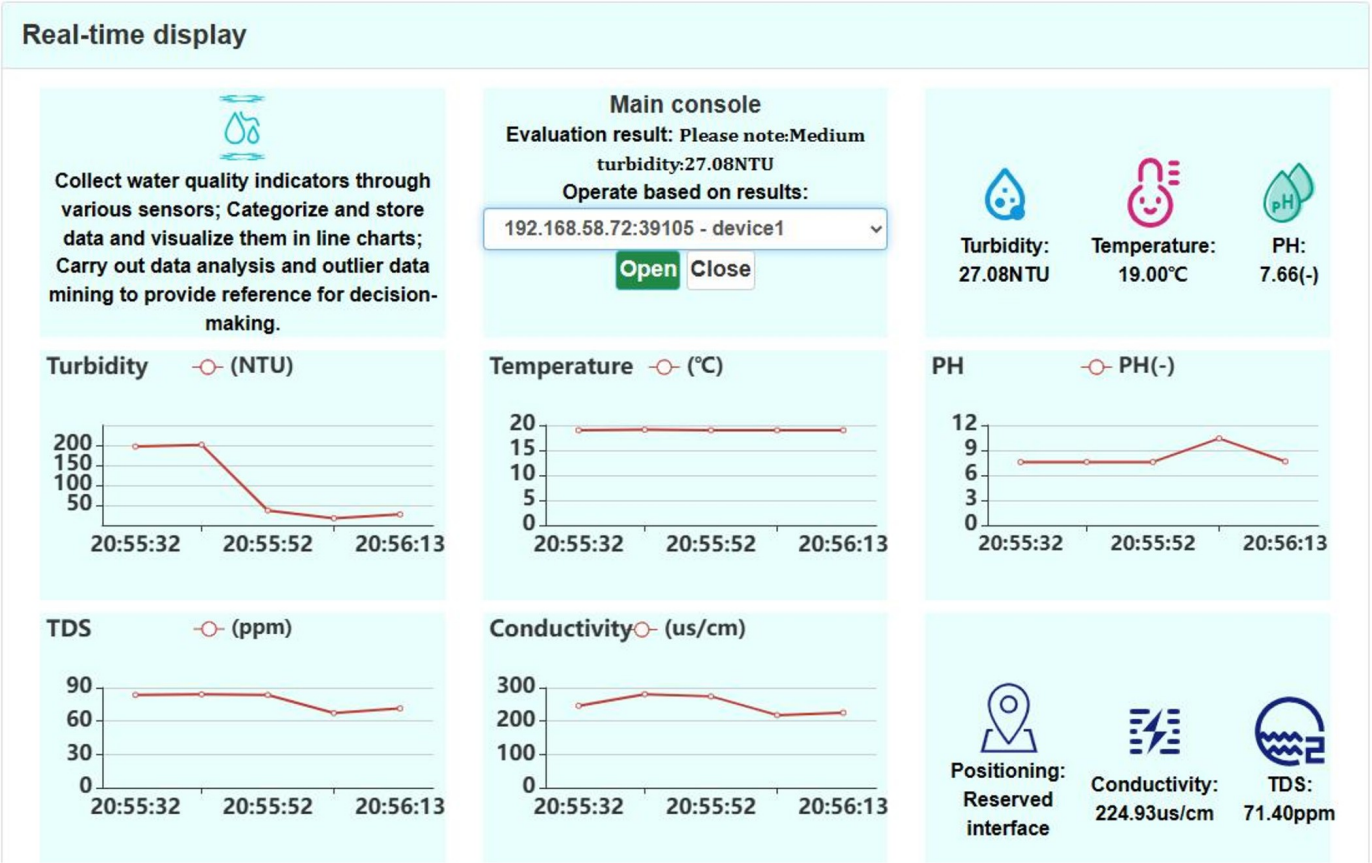
Real-time data display interface.

As depicted in Fig 10, the real-time data display interface of the system displays the current readings of turbidity, PH, temperature, conductivity, and TDS indicators alongside their respective visual line charts. To better illustrate the experimental results, we carried out additional experiments. Initially, we selected multiple water samples and immersed the probes of the five sensors in these samples. After measuring for a certain duration, we selectively removed some sensor probes and placed them in different water samples to observe the variations in different indicators, as described below.

As previously mentioned, the analysis of experimental results shows that the temperatures of various water samples remain relatively similar, resulting in temperature values that form a nearly straight line. On the other hand, there are variations in turbidity, conductivity, TDS, and pH observed among different water samples. Consequently, when measuring various water samples, the line graphs for these indicators show some degree of fluctuation.

The Main console displayed the detailed parameters of indicators, including temperature, turbidity, conductivity, TDS, and pH, along with corresponding warnings. The five sensors can monitor the data outside of range. Users can refer to them. As a result, the system can provide short-term decision-making insights based on real-time data. It is as follows.

**Fig 11:**
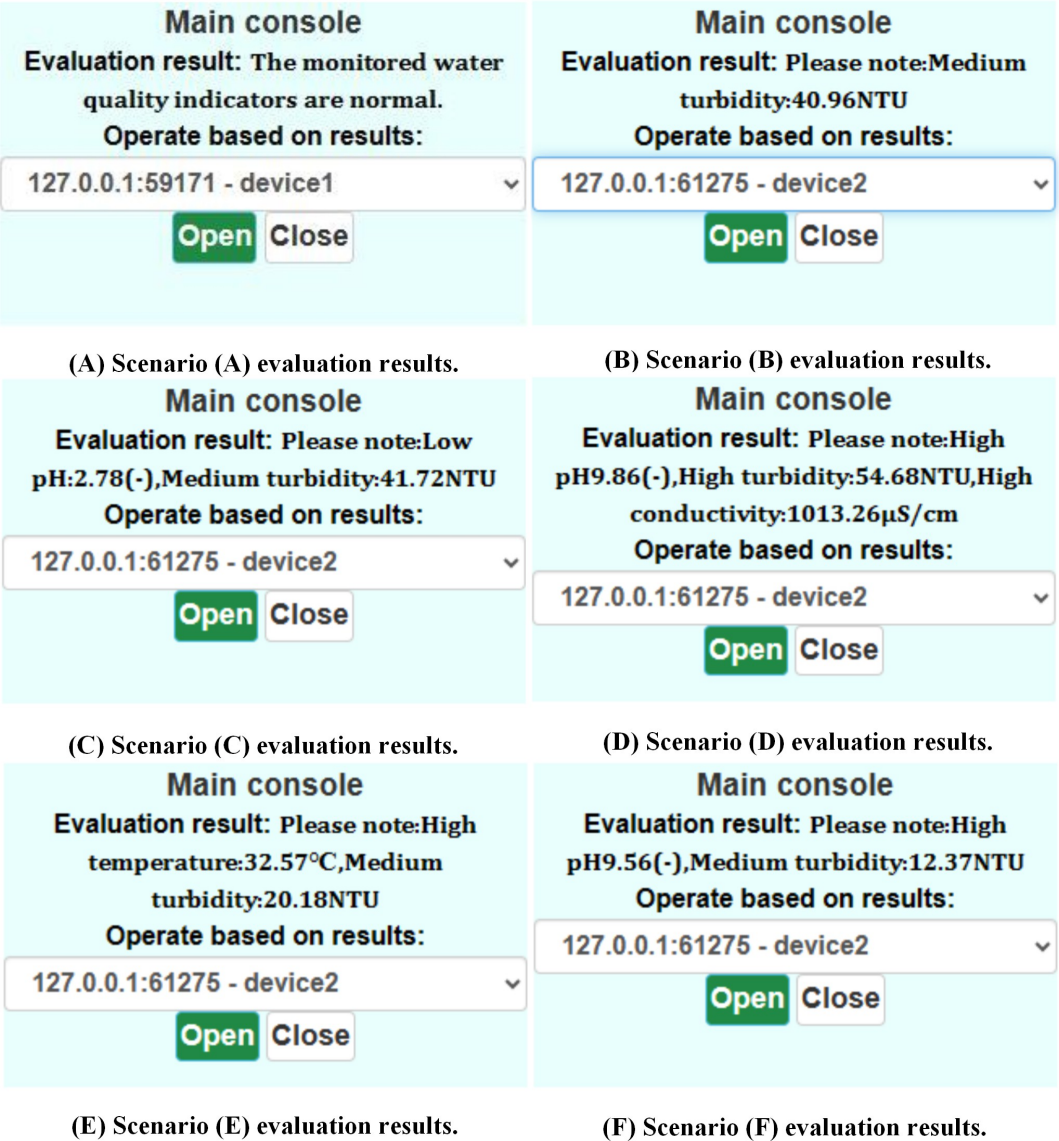
Short-term decision-making based on real-time data.

Under varying water quality conditions, the measured results vary, and the feedback from the main console also varies. Fig 11 depicts six different scenarios. For each measured scenario, the values of the indicators are shown in the Table 11 below.

**Table 11:** The values of the indicators for measured scenarios.

Scenario	Turbidity	Temperature	PH	TDS	Conductivity
**(A)**	3.45	20.57	7.78	10.49	5.21
**(B)**	40.96	20.57	7.19	10.49	5.21
**(C)**	41.72	20.57	2.78	10.49	5.21
**(D)**	54.68	20.57	9.86	500.69	1013.26
**(E)**	20.18	32.57	7.41	50.48	101.17
**(F)**	12.37	22.57	9.56	50.48	101.17

1. All indicators were within the normal range when the system identified the values in Table 11 corresponding to scenario (A). The prompt shown in Fig 11A was: "The monitored water quality indicators are normal.".
2. The turbidity was moderate when the system identified the values shown in Table 11 corresponding to scenario (B). The prompt shown in Fig 11B was: "Please note: Medium turbidity: 40.96NTU".
3. The pH was relatively low, and the turbidity was moderate when the system identified the values in Table 11 for scenario (C). The prompt shown in Fig 11C was: "Please note: Low pH: 2.78(-), Medium turbidity: 41.72NTU".
4. In case (D) of Table 11, the turbidity, pH, and conductivity were relatively high. As shown in Fig 11D, the system prompted, "Please note: High pH 9.86(-), High turbidity: 54.68NTU, High conductivity: 1013.26μS/cm".
5. The temperature was high, and the turbidity was moderate when the system identified the values in Table 11 for scenario (E). As shown in Fig 11E, the system prompted, "Please note: High temperature: 32.57°C, Medium turbidity: 20.18 NTU".
6. The pH was relatively high, and the turbidity was medium when the system detected the values listed in Table 11 for scenario (F). As shown in Fig 11F, the system prompted, "Please note: High pH 9.56(-), Medium turbidity: 12.37NTU".

As demonstrated above, the user can make decisions based on the information prompted about abnormal water quality. For instance, when the system indicates a rise in pH, we can promptly analyze and enhance supervision. In addition, we have set aside two system interface buttons, labeled "Open" and "Close," the click of which can cause the hardware side of the signal, allowing for future system expansion.

In addition to being designed for scalability, the system includes a positioning module and other indicator interfaces that can be utilized for future development.

### Historical and outlier data analysis

**Fig 12:**
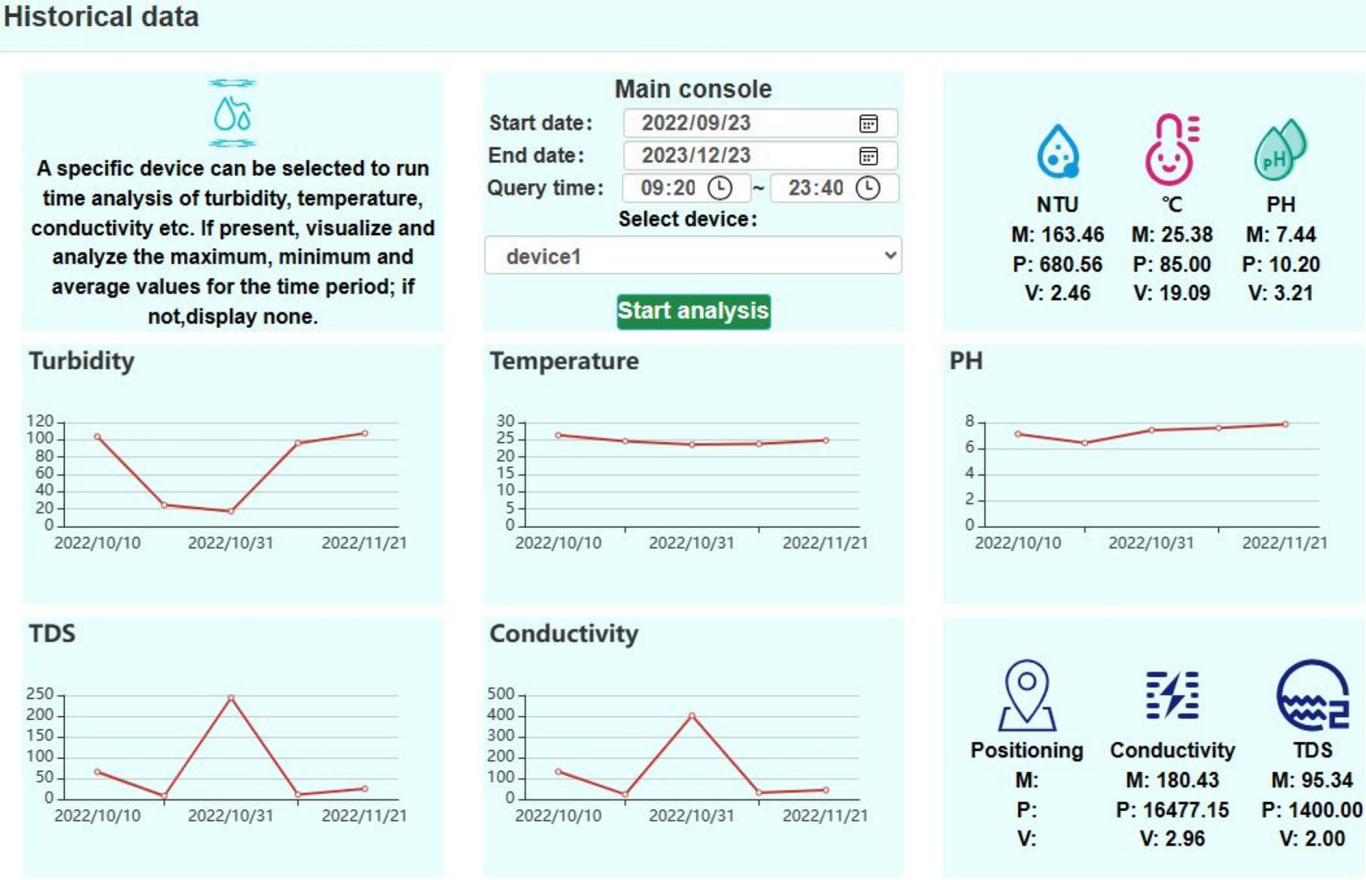
Historical data management interface.

It is important to note that our testing period extends from September 2022 to December 2023, resulting in a comprehensive dataset of approximately 100,000 data points, which serves as the basis for long-term data analysis and informed decision-making. Users can freely choose the precise historical time range, device names, and other pertinent characteristics for historical data analysis. For example, users can click on the query to view the relevant data when selecting a date range from September 2022 to December 2023, a period of approximately one year and three months, as illustrated in the Fig 12 of Main console module below. We can also click on the input box to select a different date range and devices.

After analysis, it was found that the highest amount of data was recorded in October and November during the second half of 2022, as depicted in the line chart of Fig 12 above. The line chart displays three dates: October 10th, October 31st, and November 21st, 2022. Referring to the line chart, you can observe the trend of water quality indicators, with P, V, and M representing the maximum value, minimum value, and mean value of the indicators, respectively.

The visual representation of historical data through line charts enhances our understanding of data trends. The detailed analysis is shown below.

**1. Custom time range and visual analysis:** Line charts were used to display the data retrieved by the system over a six-month period. The three annotated dates on the graph indicate that the bulk of the data is concentrated between October and November 2022. This aligns with the actual data collection scenario.

**2. Five indicator analysis:** During this period, the turbidity graph depicts a decrease followed by an increase. This can be explained by the fact that multiple water samples were measured. Initially, the samples had higher turbidity, followed by reduced turbidity, and finally, higher turbidity values again. The transition from summer to winter is evident in the overall slight decrease in temperature, which aligns with the anticipated temperature variations. The line graph’s pH indicator shows that most of the samples fall within the range of 6 to 8, indicating stable acidity-alkalinity and compliance with the minimum standards. Indicators of conductivity and total dissolved solids (TDS) exhibit similar trends when monitoring the same sample. Due to the different measuring ranges of the two sensors, they each contribute to measuring different water samples in the study.

**Fig 13:**
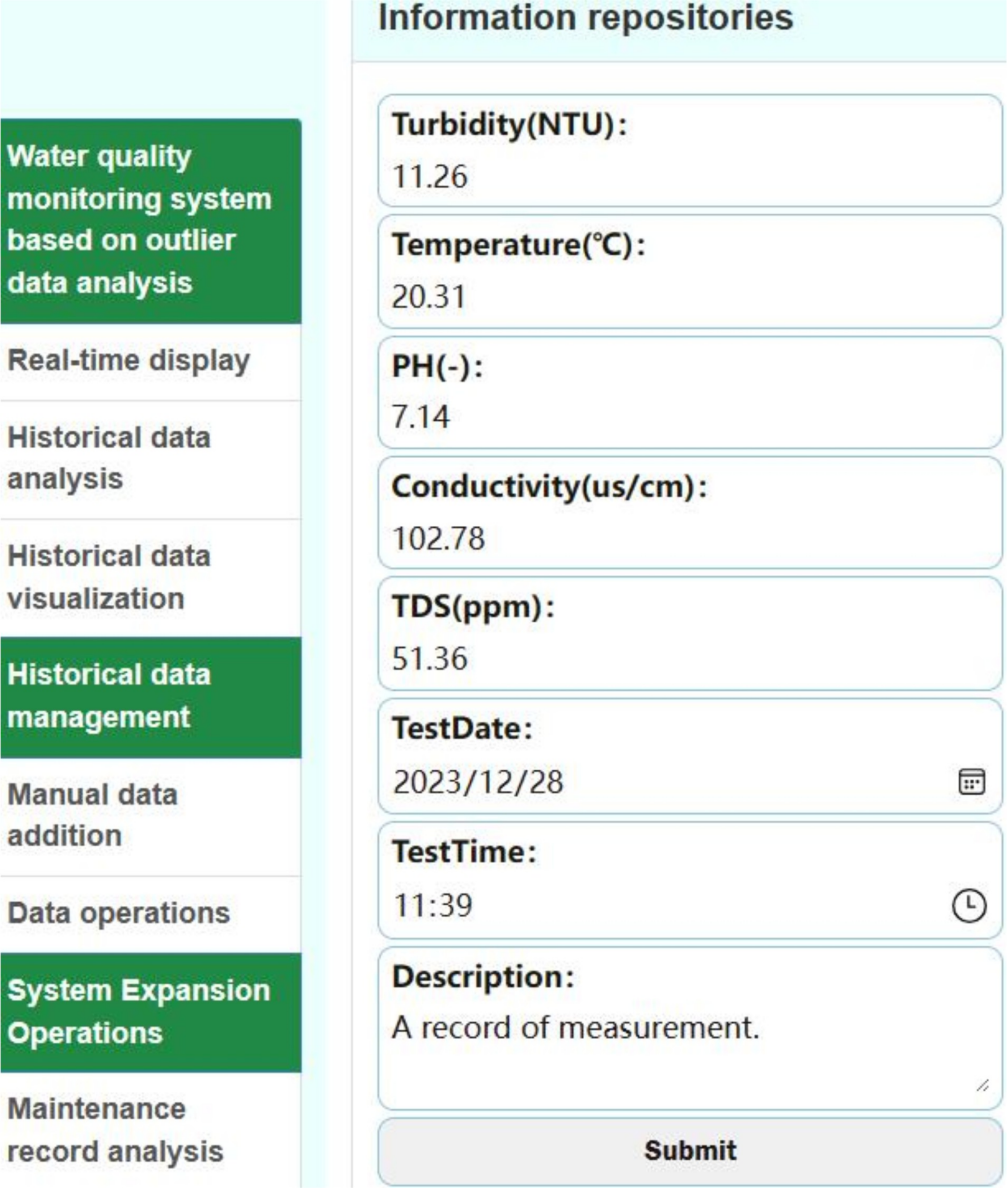
Data addition interface.

**3. Analysis of maximum, minimum, and mean values:** The system provides the maximum (P), minimum (V), and mean (M) values for the five sensor indicators within the requested time frame. For example, during that period in Fig 13, the testing of water with high conductivity yielded a maximum conductivity value of 16477.15. The maximum temperature is 85.00. This issue arose due to a coding oversight during hardware debugging, and subsequent improvements have been implemented. It will be analyzed in the anomaly data analysis module. Extreme values can act as indicators of unusual water quality conditions in future measurements, aiding in decision-making.

Overall, the system provides detailed historical data analysis results that enable users to understand data trends. The system also offers a positioning interface for easy expansion based on the maximum, minimum, and mean values of the data.

As depicted in Fig 13, the data measured by the national standard method can be added to the system to facilitate data analysis and comparison.

**Fig 14:**
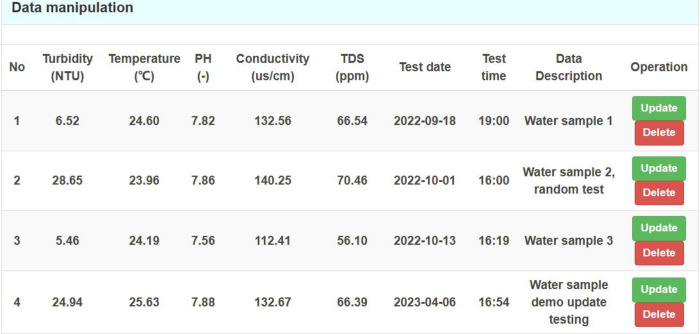
Data operation interface.

As depicted in Fig 14, the data operation interface features a "Delete" and "Modify" option which can be utilized to remove or alter data. Selecting the "Delete" option triggers a prompt that seeks reconfirmation, thereby preventing unintended deletions.

To expedite the verification of the system’s measurement effectiveness, the measurement frequency has been set to once per second, with the option to adjust this parameter according to future deployment requirements. After several months of testing, the system has accumulated over 100000 data points. Five distinct time periods from November 2022 to December 2023 were selected for the analysis of outlier data. We can refer to Table 9 for a comprehensive overview of the data. The quartile method was utilized to identify outlier values and compute the proportion of outlier data, as illustrated in Table 12 below.

**Table 12:** Outlier data proportion table.

Data No	Outlier data proportion (%)				
	Turbidity (NTU)	PH (-)	Temperature (°C)	Conductivity(us/cm)	TDS (ppm)
1	3.09	0.73	0.29	2.97	1.51
2	3.11	0.68	0.43	2.92	1.24
3	3.02	0.82	0.82	2.73	0.98
4	3.24	0.76	0.12	2.92	1.36
5	3.07	1.19	0.45	3.07	1.13
Mean	3.11	0.84	0.42	2.92	1.24

The analysis indicates that the system is stable when measuring PH, temperature, and TDS indicators with a relatively low proportion of outlier data. When measuring turbidity and conductivity indicators, the proportion can also be kept within 5%. A detailed analysis of the turbidity and conductivity indicators, which exhibit a greater proportion of outlier data, was undertaken. Table 13 identifies some outlier values of conductivity.

**Table 13:** Partial outlier data table.

Data type	Data No	Outlier data analysis								
		Mean value	Partial Outlier data							
**Conductivity**	1	126.86	100.07	113.52	146.38	142.91	142.73	98.97	100.56	92.96
	2	34.65	0.00	104.11	65.26	85.43	6.14	65.39	58.85	78.32
	3	63.71	15.23	25.96	31.36	210.02	21.51	96.92	15.23	25.96
	4	193.33	223.91	160.96	123.3	102.68	162.68	142.29	224.21	236.83
	5	399.92	300.68	356.36	441.04	363.48	494.01	449.1	480.96	330.85
**Turbidity**	1	100.11	79.83	78.82	72.85	139.56	79.53	76.47	77.95	136.46
	2	31.16	50.56	16.87	18.87	18.53	10.22	83.21	56.57	58.46
	3	129.53	155.00	173.35	171.96	144.75	101.5	154.51	156.61	155.21
	4	264.29	200.17	208.56	200.66	302.05	301.35	229.96	339.26	223.26
	5	163.60	142.40	141.01	145.16	142.83	141.87	181.57	131.46	189.94
**Temperature**	1	25.51	82.00							

Table 13 presents a detailed analysis of the indicators, focusing on turbidity and conductivity, which exhibit a higher prevalence of outlier data. It also focuses on temperature, which has less frequent outlier data. The third column depicts the mean value, and the following columns depict the detected outliers. The system shows a relatively low occurrence of outlier data. Taking Data No 1 in the conductivity indicator as an example, during this period, the mean value is 126.86, with 8 outlier data points identified. Analyzing the data enables us to pinpoint the reasons for the occurrence of outliers, facilitating subsequent improvements. It is shown below.

**1. Temperature indicator:** The temperature indicator has a few outlier data points. The system failed to reset the data point "82.00" to 0 during hardware debugging, leading to inaccurate data. The subsequent upgrades have resolved this issue.

**2. Conductivity and turbidity sensor:** Positioning changes during measurements can disrupt the sensors. Hardware adjustments, such as adding more fixtures, can be made to address the issue and improve stability. Accuracy could be further improved by considering the average data over a specific time as a reference value. More accurate data collection could be achieved by allowing the sensor to adjust before transmitting data when switching measurement samples.

After the identification of outlier data, seven techniques are utilized to rectify the data, including mode filling, mean filling, median filling, Lagrange interpolation filling, k-nearest neighbor filling, utilization of the previous non-empty element filling, and direct deletion of outlier data. The variance index is utilized to measure the effectiveness of the techniques, as depicted below.

**Fig 15:**
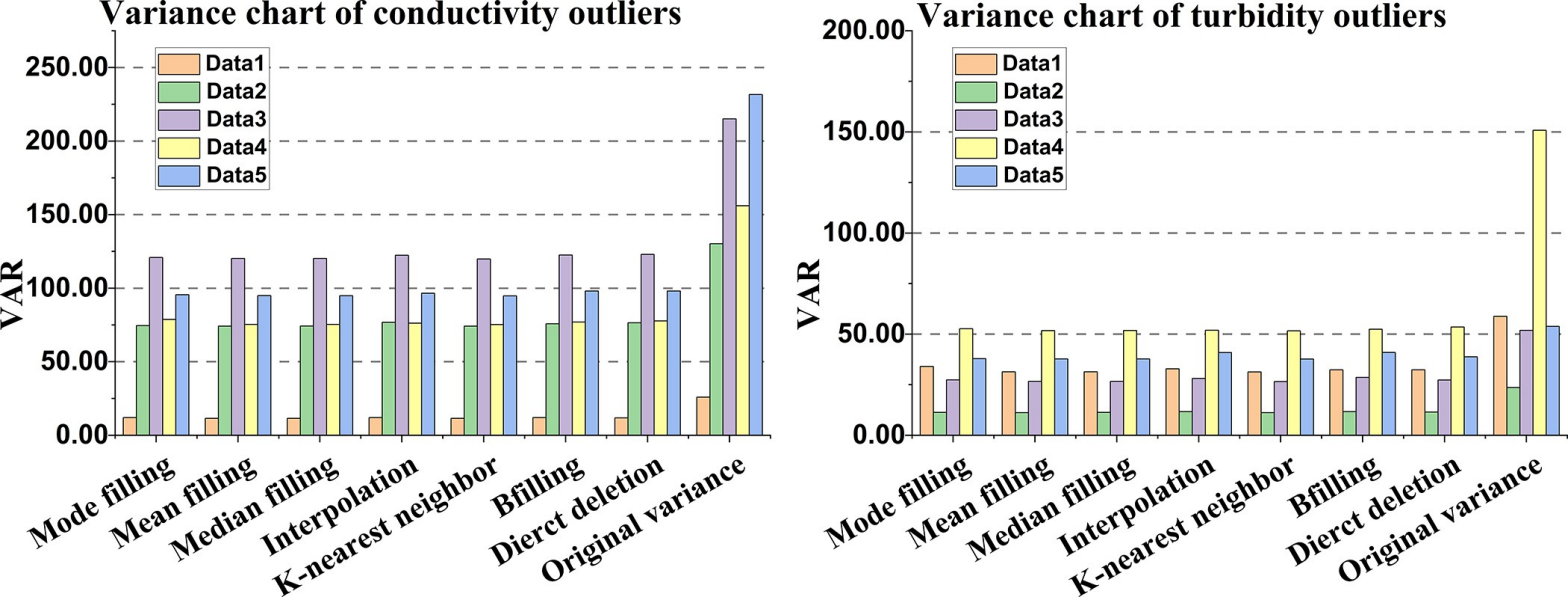
Variance chart of indicator outlier data. (A) Variance table of conductivity outlier data, (B) Variance table of turbidity outlier data.

Fig 15A and 15B depict the variance tables of outlier data for conductivity and turbidity, respectively. The x-axis represents the seven methods, while the y-axis denotes the data variance value. The rightmost column chart illustrates the original variance of the five data, while the 7-cluster column chart on the left depicts the variance obtained after processing the data from five time periods using seven methods. The specific variance value of conductivity is shown in Table 14 below.

**Table 14:** Variance value of conductivity.

Indicators	Methods	Data No				
		Data1	Data2	Data3	Data4	Data5
**Conductivity**	Mode filling	12.04	74.6	120.84	78.85	95.64
	Mean filling	11.53	74.28	120.22	75.42	94.98
	Median filling	11.53	74.35	120.27	75.46	94.95
	Interpolation	12.12	76.84	122.41	76.26	96.63
	K-nearest neighbor	**11.51**	**74.23**	**119.89**	**75.32**	**94.83**
	Bfilling	12.04	75.9	122.62	77.09	98.12
	Dierct deletion	11.88	76.51	123.04	77.74	98.13
	Original variance	25.94	130.32	215.14	156.07	231.71
**Turbidity**	Mode filling	34.08	11.37	27.42	52.71	37.88
	Mean filling	31.4	11.22	26.7	51.78	37.77
	Median filling	31.39	11.37	26.69	51.83	37.78
	Interpolation	32.88	11.7	28.12	51.97	40.92
	K-nearest neighbor	**31.35**	**11.2**	**26.61**	**51.66**	**37.72**
	Bfilling	32.38	11.71	28.65	52.49	40.96
	Dierct deletion	32.36	11.57	27.35	53.59	38.88
	Original variance	58.85	23.64	51.89	150.83	53.83

"Bfilling" in Table 14 refers to using the value of the previous non-empty element to fill the current one. The analysis of the Fig 15A and 15B, as well as Table 14, are as follows.

When the system measures turbidity and conductivity, the variance is also different. The smaller the variance is, the more stable it is. Compared with the original variance on the right, the data variance is significantly decreased using seven methods. The shape of the seven clusters on the left is similar, which indicates that the seven methods can effectively handle outliers and decrease the overall variance. The analysis of Table 14 shows that although the seven methods can effectively reduce the overall variance, the K-nearest neighbor filling method is more effective compared with others. Using the K-nearest neighbor approach, the filled data has a comparatively low variance. Finally, the reasons for the outlier data of turbidity and conductivity are summarized. The turbidity sensor is susceptible to fluctuations due to ambient light, whereas the conductivity sensor is susceptible to positional vibrations during measurement.

On the one hand, applicable hardware materials can be manufactured to reduce the environmental impact; on the other hand, the mean data over a given period can be chosen as the indicator’s reference value. In addition, each outlier management method has a unique application scenario and can be implemented based on specific requirements.

### Data-assisted decision-making

Both historical data analysis and outlier data analysis results are helpful for decisions-making. The following is an explanation of decision-making supported by data analysis.

**1.Trend analysis:** We explore the factors contributing to data trends rather than just focusing on them during monthly trend analyses. For example, to make timely regulatory decisions, if turbidity levels increase over time, we can focus on assessing whether it is linked to seasonal fluctuations, special events, etc.

**2.Analysis of outlier values:** When dealing with system-reported outliers, such as a maximum temperature value of 85.00, it is important to conduct a detailed analysis to understand the cause of these outliers. If outliers are caused by hardware or software issues, it is necessary to promptly repair and recalibrate sensors. This decision helps to improve the reliability and accuracy of the data.

**3.Creating schedules for routine maintenance and calibration:** Data analysis enables us to develop maintenance schedules. For example, we may consider increasing the frequency of temperature sensor maintenance and calibration if a higher number of temperature outliers are detected within a given time frame. The water quality monitoring system can provide more precise and reliable data by addressing instrument instability through research and increasing maintenance frequency.

**4.Making decisions with statistical data:** identify possible severe water quality conditions by analyzing statistical data, including the maximum, minimum, and mean values provided by the system. For instance, if an unusually high conductivity value is detected, more research can be done to determine if there are any exceptional circumstances related to the water quality at that time, such as industrial discharges or other sources of contamination. Subsequently, decisions can be made to enhance the monitoring of water quality and carry out suitable corrective action.

These decision-making techniques are essential for maintaining data accuracy, ensuring sensor performance, and addressing water quality issues.

### Comparative analysis of two measurement methods

Our Comparative analysis of two measurement methods accomplishes two main goals. First, we assessed the indicator values for identical water samples using the national standards-specified procedures, treating them as reference values. We used our newly developed system to measure the indicator values once again simultaneously. We met our usage requirements by determining that all indicators had measurement errors within 4% by performing a comparison analysis of measurement errors. Secondly, the comparative experiments reveal on the shortcomings in the development of the system. With the flexibility provided by the available funding, we can optimize the system to enhance its performance and improve the user experience.

An experiment was conducted utilizing data collected from specific segments of the Li River. The results are presented in Table 15, which includes five datasets: four corresponding to actual water samples and one pertaining to the standard solution. The national standard method was utilized to determine the standard value, followed by the utilization of the method implemented in the system to obtain the measured value. Repeat the measurement several times for samples and the mean value as shown below.

**Table 15:** Comparative experiment data table.

Sample No	Temperature (°C)		PH (-)		TDS (ppm)		Conductivity(us/cm)		Turbidity (NTU)	
	Measured	Standard	Measured	Standard	Measured	Standard	Measured	Standard	Measured	Standard
1	22.47	22.27	6.62	6.45	63.03	61	117.92	122	3.78	4
2	21.37	21.15	6.93	6.78	71.25	69	133.18	138	15.27	15.9
3	22.35	22.14	7.25	7.11	207.96	202	392.21	404	45.37	46.87
4	24.44	24.69	7.23	7.41	293.6	287	557.73	574	62.53	64.86
5	23.48	23.25	6.99	6.86	725.97	706.5	1375.64	1413	96.35	100

The final trial data is depicted above, with the relative error demonstrated in Table 16, based on the information outlined in Table 15.

**Table 16:** Comparison experiment error table.

Sample No	Relative error (%)				
	Temperature (°C)	PH (-)	TDS (ppm)	Conductivity(us/cm)	Turbidity (NTU)
1	0.90	2.64	3.33	3.34	5.50
2	1.04	2.21	3.26	3.49	3.96
3	0.95	1.97	2.95	2.92	3.20
4	1.01	2.43	2.30	2.83	3.59
5	0.99	1.90	2.76	2.64	3.65
Mean	0.98	2.23	2.92	3.05	3.98

Table 16 illustrated the average relative error for each indicator, with the respective values for temperature, pH, and TDS measurement averaging at 0.98%, 2.23%, and 2.92%. The remaining two indices yield average error rates of 3.05% and 3.98%, respectively. Overall, the results indicate that the average measurement error for water quality indicators can be maintained within 5%.

## Conclusion

This study describes the development of a monitoring and data analysis system for the Li River’s water quality. The system utilizes sensors to collect data on five water quality indicators and integrates sensor technology and data analysis techniques to attain efficient monitoring and data analysis. Notably, the system’s implementation results in a re-duction in the measurement relative errors for turbidity, PH, temperature, conductivity, and TDS parameters, with the average errors decreasing to 3.98%, 2.23%, 0.98%, 3.05%, and 2.92%, respectively. This study also places a heavy emphasis on data analysis, particularly the analysis of outlier data, which affects the precision of water quality data and subsequent decision-making processes. The implementation of the quartile method enables the identification of outlier values for each indicator, with the proportions for temperature, PH, turbidity, conductivity, and TDS measuring at 0.42%,0.84%,3.11%,2.92%, and 1.24%, respectively. On the cloud platform, the distribution characteristics of sensor data are analyzed and displayed interactively to facilitate routine water quality monitoring and early warning. To facilitate decision-making, by identifying unusual patterns, extreme values, and data trends, we can promptly investigate and address potential issues, ensuring the reliability of the collected data. It is valuable for making decisions related to water quality management, as it provides insights into seasonal variations and potential sources of pollution. In addition, decision-makers can utilize this information to schedule regular maintenance activities, ensuring the continuous accuracy and reliability of the sensor network.

In addition to the indicators examined in this study, additional indicators of water quality will be the subject of future research. To accomplish this, the system is designed with reserved interfaces that can be used for future development. By expanding on this study’s findings and integrating additional sensors and algorithms, it is possible to rapidly implement data monitoring and analysis for other water quality indicators.
